# Evaluation of Low-Dose Radiation Treatment Effects Using Conductivity, Diffusivity, and Brain Tissue Volumes Treated in Patients with Mild Alzheimer’s Disease: Exploratory Investigation

**DOI:** 10.3390/diagnostics16081163

**Published:** 2026-04-14

**Authors:** Weon Kuu Chung, Hwang Mi Kim, Mun Bae Lee, Kisoo Kim, Oh-In Kwon, Ye Jin Yoo, Hak Young Rhee, Geon-Ho Jahng

**Affiliations:** 1 Department of Radiation Oncology, Kyung Hee University Hospital at Gangdong, Kyung Hee University College of Medicine, Seoul 05278, Republic of Korea; wkchung16@gmail.com (W.K.C.); yejinyoo1@gmail.com (Y.J.Y.); 2 Department of Biomedical Engineering, College of Electronics and Information, Kyung Hee University, Gyeonggi-do 17104, Republic of Korea; 3 Department of Mathematics, College of Basic Science, Konkuk University, Seoul 05029, Republic of Korea; munbael@konkuk.ac.kr (M.B.L.);; 4 Department of Neurology, Kyung Hee University Hospital at Gangdong, Kyung Hee University College of Medicine, Seoul 05278, Republic of Korea; 5 Department of Radiology, Kyung Hee University Hospital at Gangdong, Kyung Hee University College of Medicine, Seoul 05278, Republic of Korea

**Keywords:** low-dose radiation therapy (LDRT), Alzheimer’s disease, high-frequency conductivity (HFC), diffusion tensor imaging (DTI), quantitative brain MRI

## Abstract

**Purpose**: No prior clinical studies have quantitatively evaluated the effect of low-dose radiation therapy (LDRT) on Alzheimer’s disease (AD) brain changes using multi-modal MRI. This study examined the feasibility of using conductivity, diffusion, and brain tissue volume measures to detect treatment effects in patients with AD receiving LDRT. **Methods**: Nine patients with mild AD were enrolled in three groups. Three patients in each group were assigned to the control group (0 cGy) and the treated groups [24 cGy/6 fractions (4 cGy for each fraction) and 300 cGy/6 fractions (50 cGy for each fraction)]. Conductivity, diffusivity, and brain tissue volume were acquired at baseline and 6 months post-treatment and were evaluated to assess within-group MRI changes and evaluate associations between MRI measures and Mini-Mental State Examination (MMSE) scores. **Results**: Region-of-interest (ROI) analyses identified substantial changes in high-frequency conductivity (HFC) (e.g., left insula), cerebrospinal fluid (CSF) volumes (e.g., anterior cingulate, limbic regions), and diffusion tensor imaging (DTI) metrics, such as axial diffusivity (AxD) and fractional anisotropy (FA), in fusiform, thalamic, hippocampal, and occipital areas. Correlation analysis showed strong associations between MRI measures and cognition, most notably HFC in the left fusiform gyrus (r = 0.843, *p* = 0.0043) after treatment. Diffusion indices across multiple regions also showed significant positive or negative correlations with MMSE. **Conclusions**: This exploratory clinical study demonstrates that LDRT induces measurable physiological and microstructural alterations in the brain detectable via conductivity and diffusion MRI. Conductivity emerged as the sensitive biomarker, showing strong cognitive correlations. These exploratory findings suggest that multi-modal quantitative MRI can serve as an effective tool for evaluating treatment response in clinical LDRT for AD.

## 1. Introduction

Alzheimer’s disease (AD) is a progressive neurodegenerative disorder that primarily affects memory, thinking, and behavior. The accumulation of extracellular amyloid plaques and intracellular neurofibrillary tangles in the brain constitutes the core pathophysiological features of Alzheimer’s disease and is associated with neuronal cell death [1]. In addition, recent studies have demonstrated that neuroinflammation plays an important role in the progression of AD [2]. Risk factors for AD include age (most people with Alzheimer’s are 65 or older), family history, genetics, certain lifestyle factors, such as lack of exercise, smoking, and poor diet, and other medical conditions, like hypertension, diabetes, and high cholesterol. Current treatment options primarily focus on alleviating the clinical symptoms of the disease. Recently, a drug for disease-modified treatment was developed and used to target the underlying neuropathological and pathophysiological mechanisms. Ongoing research is focused on understanding the underlying causes of AD, developing new treatments, and finding ways to prevent it. Therefore, there is a demand for developing new non-drug therapeutic methods in addition to pharmacological therapy.

Whole-brain radiotherapy has been widely employed in the management of various brain disorders, particularly in patients with multiple brain metastases. Beyond its established oncologic applications, radiation therapy has also been used in selected non-malignant conditions, including systemic amyloidosis, where anti-inflammatory and modulatory effects have been reported [3,4,5]. Pilot clinical studies have recently explored LDRT as a potential treatment for AD, centered on the hypothesis that low-dose irradiation may attenuate neuroinflammation and oxidative stress. Given that neuroinflammation is a critical driver of AD pathology, LDRT may modulate the immune response and suppress chronic inflammatory pathways within the central nervous system.

Experimental studies have suggested that radiation therapy may exert biological effects relevant to the pathophysiology of AD, particularly when delivered at low doses. Low-dose irradiation has been reported to disrupt hydrogen bonding within amyloid plaques [6], increase blood–brain barrier (BBB) permeability to facilitate monocyte trafficking and amyloid clearance, and modulate microglial activation toward an M2 phenotype, which has been associated with tissue repair and regenerative processes [7,8]. In preclinical studies using animal models of AD, low-dose radiation therapy (LDRT), typically administered at doses substantially lower than those used in conventional oncologic radiotherapy and often delivered in a fractionated manner, has been shown to reduce amyloid-β plaque burden. These findings suggest that the biological effects of low-dose irradiation may differ fundamentally from those observed at higher therapeutic doses used for malignancies.

Based on these laboratory and animal data, early-phase clinical investigations have been initiated to evaluate radiation therapy as a potential therapeutic approach for AD. In these studies, LDRT has generally been administered as whole-brain irradiation using low total doses delivered over multiple fractions to minimize toxicity while aiming to achieve immunomodulatory effects. Such clinical trials are currently planned or ongoing in several countries, including the United States, Switzerland, and South Korea (ClinicalTrials.gov identifiers: NCT02769000, NCT03352258). More recently, pilot clinical studies have explored LDRT as a treatment strategy for AD based on the hypothesis that low-dose, fractionated irradiation may attenuate neuroinflammation and oxidative stress in the brain. The proposed therapeutic rationale of LDRT in AD centers on its immunomodulatory properties, particularly the regulation of chronic inflammatory pathways. Given that neuroinflammation is recognized as a key component of AD pathology, low-dose, fractionated radiation may contribute to disease modulation through the reduction in sustained inflammatory processes within the central nervous system.

MRI has been used to evaluate treatment effects in clinics. First, 3D T1-weighted MRI is commonly used to assess brain tissue volume parameters such as gray matter volume (GMV), white matter volume (WMV), and cerebrospinal fluid (CSF) volume (CSFV). Second, ionic conductivity is a physical parameter influenced by the movement of charges in response to an electric field. Conductivity escalates with increasing ion concentrations and ionic mobility. Magnetic resonance electrical properties tomography (MREPT) is a noninvasive technique for mapping conductivity, which leverages standard MRI systems without the need for external electrodes or currents. It enables the imaging of conductivity at Larmor frequencies (approximately 128 MHz for a 3 Tesla MRI system), a parameter also known as high-frequency conductivity (HFC) [9]. This electrical property is sensitive to pathological and physiological conditions [10,11]. In addition, recent studies demonstrated that the conductivity value was a sensitive index to evaluate the effect of high-dose radiation treatment in mouse brain tumors [12]. However, those studies were performed with high-dose radiation irradiation with very short-term evaluation after irradiation. Finally, diffusivity quantifies the displacement of water protons over time. Diffusion Tensor Imaging (DTI) is a tensor model to measure and visualize the diffusion of water molecules in biological tissues, particularly in the brain. This technique provides isotropic and anisotropic diffusivity. Previous studies demonstrated that the diffusion coefficient was a sensitive index for evaluating the effect of radiation treatment in mouse brain tumors [12].

Preclinical models suggest that whole-brain radiotherapy may facilitate the clearance of amyloid-β plaques and provide neuroprotection. While the exact mechanisms remain under investigation, a few pilot studies have evaluated the safety and clinical efficacy of LDRT in humans, primarily focusing on cognitive outcomes. However, quantitative MRI-based evaluations of these effects remain unreported. Therefore, in this study, we first reported the results of LDRT in AD patients using conductivity, diffusivity, and brain tissue volume parameters. Although this was a very preliminary study, it is the first quantitative evaluation using multi-modal parameters in clinical LDRT in AD patients.

## 2. Materials and Methods

### 2.1. Participants

#### 2.1.1. Ethical Approval

This study was conducted in accordance with a protocol approved by the Institutional Review Board (IRB) of Kyung Hee University Hospital at Gangdong (khnmc2022-03-030, approved date: 10 May 2022). It adhered to the Good Clinical Practice (GCP) guidelines established by the Ministry of Food and Drug Safety and the International Council for Harmonization (ICH). Written consent was received from both the patient and the legal participant’s family. The detailed description of this clinical protocol was published elsewhere [13].

#### 2.1.2. Inclusion Criteria

Participants were eligible for inclusion if they met all of the following criteria: (1) a diagnosis of Alzheimer’s-type dementia and stable treatment with conventional anti-dementia medication for at least 3 months before enrollment; (2) confirmed cerebral amyloid accumulation as demonstrated by amyloid positron emission tomography (PET); (3) mild to moderate dementia, defined by a Mini-Mental State Examination (MMSE) score of 13–24 and a Clinical Dementia Rating (CDR) score of 0.5 or 1; (4) age between 60 and 85 years; (5) ability to undergo cognitive function assessments and imaging studies required by the study protocol; (6) availability of a caregiver capable of providing reliable information regarding the participant’s overall condition, cognitive status, and functional changes; and (7) provision of written informed consent by the participant or their legally authorized representative.

#### 2.1.3. Exclusion Criteria

Participants were excluded if any of the following criteria were met: (1) prior history of cranial radiation therapy; (2) history of seizure disorders; (3) active dermatologic disease involving the scalp; (4) history of malignant tumors; (5) pregnancy or breastfeeding; or (6) any other clinically significant condition for which the investigator judged participation in the study to be inappropriate. The detailed design of this clinical trial is described in the Supplementary Materials.

### 2.2. Radiation Dose and Treatment Duration

Whole-brain radiotherapy was delivered using a linear accelerator with 6-MV photon energy (Varian Clinac IX, Varian Medical Systems, Palo Alto, California, USA). Two experimental dose levels and one sham control were employed. The selected doses (24 cGy and 300 cGy) were substantially lower than those used in conventional oncologic whole-brain radiotherapy and were, therefore, considered unlikely to induce radiation-related cognitive decline [3]. Participants in the control group (A) received sham whole-brain radiotherapy following the same treatment schedule (six sessions over 3 weeks) with a total dose of 0 cGy. Participants in experimental group 1 (B) received a total dose of 24 cGy, administered as 4 cGy per fraction, twice weekly over six sessions across 3 weeks. This regimen was informed by a prior case report suggesting cognitive improvement following repeated low-dose cranial exposure [14]. Participants in experimental group 2 (C) received a total dose of 300 cGy, administered as 50 cGy per fraction, twice weekly over six sessions across 3 weeks. This dose was based on preclinical studies demonstrating reduced amyloid plaque accumulation and modulation of neuroinflammation in AD models [15].

This paper presents results from a single-center exploratory study, as advanced MRI techniques were performed exclusively at this institution. Table 1 summarizes the demographic characteristics of the three groups. In this study, we only reported data obtained from baseline and 6 months after treatment. Each group had three participants. Participants were assigned to either the sham control or one of two LDRT dose groups according to the pilot study scheme; notably, allocation was not randomized, and the study did not employ a formal blinded design. Consequently, selection and assessment bias cannot be entirely excluded, and these findings should be interpreted as hypothesis-generating.

### 2.3. Brain MRI Acquisition

The standard MRI protocol was included in a sagittal 3D T1-weighted (3D T1W) image, axial T2-weighted turbo-spin-echo image, and axial FLAIR image. This protocol was performed in multiple institutes. Brain MRI scans were conducted at baseline, 6 months, and 12 months after radiation treatment. In addition, only one institute performed the advanced imaging techniques that are reported in this paper with the data acquired at the baseline and at 6 months after treatment. MRI was performed using a 3.0 Tesla MRI system equipped with a 32-channel sensitivity encoding head coil (Ingenia, Philips Medical System, Best, The Netherlands).

#### 2.3.1. Strucrural Imaging

A sagittal structural 3D T1W image was acquired with the fast field-echo (FFE) sequence for image registration and brain tissue segmentation with a voxel size of 1 × 1 × 1 mm^3^. In addition, T2-weighted turbo-spin-echo and fluid-attenuated inversion recovery (FLAIR) images were also acquired to evaluate any brain abnormalities.

#### 2.3.2. MREPT Imaging

A six-echo turbo-spin-echo (TSE) pulse sequence was used for the brain MREPT images [11] with the repetition time (TR) = 3200 ms, first echo time (TE) = 12 ms with 12 ms intervals, number of slices = 20 without a gap between the slices, slice orientation = transverse, and acquired voxel size = 2 × 2 × 5 mm^3^. This is a multi-echo sequence. Therefore, the TSE factor was 6, indicating that the number of echoes was 6. The scan time of the MREPT sequence was 6 min and 5 s. Real and imaginary images were saved to reconstruct the conductivity map.

#### 2.3.3. DTI Imaging

A single-shot spin-echo echo-planar imaging (SS-SE-EPI) pulse sequence was used to obtain diffusion tensor imaging with two *b*-values of nominally 1000 and 2000 s/mm^2^, using 16 and 32 diffusion tensor gradient directions, respectively. The imaging parameters were TR/TE = 15,000/86 ms and voxel size = 2 × 2 × 2 mm^3^. Total scan times were 2 min 15 s, 4 min 45 s, and 8 min 45 s for b-values of 0, 1000, and 2000 s/mm^2^, respectively.

### 2.4. Reconstruction of Quantitative Maps

#### 2.4.1. Conductivity Mapping

The relationship between the B_1_ field, denoted as $B_{1}$, and the electrical properties (conductivity and permittivity) is expressed as

(1) $$\nabla^{2}B_{1}=i\omega \mu_{0}\;\tau_{H}B_{1}\;-\frac{\nabla \tau_{H}}{\tau_{H}}\times \left(\nabla \times B_{1}\right)$$

where *ω* is the angular frequency, $\mu_{0}=4\pi \times 10^{-7}\;N/A^{2}$ is the magnetic permeability of the free space, and $\tau_{H}=\sigma_{H}+i\omega \epsilon_{H}$ at high-frequency conductivity $\sigma_{H}$ and permittivity $\epsilon_{H}$ [9,16]. The transverse field of $B_{1}$ can be decomposed into the positively rotating field $B_{1}^{+}$ and the negatively rotating field $B_{1}^{-}$. We denoted $\phi^{+}$ and $\phi^{-}$ as the phase terms of $B_{1}^{+}$ and $B_{1}^{-}$, respectively. By assuming $\sigma_{H}\gg \omega \epsilon_{H}$, a phase-based MREPT formula was derived as

(2) $$\left(\nabla \phi^{tr}\cdot \nabla \left(\frac{1}{\sigma_{H}}\right)\right)+\frac{\nabla^{2}\phi^{tr}}{\sigma_{H}}-2\omega \mu_{0}=0$$

where $\phi^{tr}=\phi^{+}+\phi^{-}$ is the so-called transceive phase, which can be obtained using a conventional scanner. To stabilize the formula [2], the MREPT formula based on a convection reaction equation can be derived by adding the regularization coefficient c  [17]:

(3) $$-c\nabla^{2}\left(\frac{1}{\sigma_{H}}\right)+\left(\nabla \phi^{tr}\cdot \nabla \left(\frac{1}{\sigma_{H}}\right)\right)+\frac{\nabla^{2}\phi^{tr}}{\sigma_{H}}=2\omega \mu_{0}$$

When selecting regularization coefficients, there is a trade-off between solution accuracy and stability. The choice for the regularization coefficients c=0.03 was made empirically from simulation data and studies [11,17].

MREPT depends upon the relatively weak phase signal of a secondary RF magnetic field from the induced electrical current by the time-varying RF field. Due to the weak phase signal and noise artifacts, a multi-echo spin-echo MR pulse sequence is advantageous to reduce the noise artifacts using the weight for the *k*th echo:

$$\phi^{tr}=\sum_{k=1}^{N_{E}}w_{k}\phi^{k},\;w_{k}=\frac{{\left|\rho_{k}\right|}^{2}}{\sum_{j=1}^{N_{E}}{\left|\rho_{j}\right|}^{2}}$$

where $\phi^{k}$ and $\rho_{k}$ are the phase signal and complex MR signal, respectively, for the *k*-th echo. To solve the convection reaction partial differential equation in (3), we used the 2-dimensional finite-difference method. For each image matrix, Equation (3) is written as

(4) $$\left[\begin{array}{c} \vdots \\ -c\left(\frac{\partial^{2}}{\partial x^{2}}+\frac{\partial^{2}}{\partial y^{2}}\right)+\frac{\partial \phi^{tr}}{\partial x}\frac{\partial }{\partial x}+\frac{\partial \phi^{tr}}{\partial y}\frac{\partial }{\partial y}+\frac{\partial^{2}\phi^{tr}}{\partial x^{2}}+\frac{\partial^{2}\phi^{tr}}{\partial y^{2}}\\ \vdots \end{array}\right]\left[\begin{array}{c} \vdots \\ \frac{1}{\sigma_{H}}\;\\ \vdots \end{array}\right]=\left[\begin{array}{c} \vdots \\ 2\omega \mu_{0}\\ \vdots \end{array}\right]$$

The finite-difference method for solving Equation (4) is to find the solutions of a linear matrix system Ax = b with the appropriate processing of the Dirichlet boundary conditions. A is a staff matrix, $x=(\frac{1}{\sigma_{H1}},\frac{1}{\sigma_{H2}},\ldots ,\frac{1}{\sigma_{HN}})$, and $b=(2\omega \mu_{0},\;2\omega \mu_{0},\;\ldots ,\;2\omega \mu_{0})$, respectively. We used the finite-difference method to solve the above matrix system with a regularization coefficient c=0.03 in Equation (4) [11].

#### 2.4.2. DTI Mapping

A 3 × 3 diffusion tensor with three eigenvalues ($\lambda_{1}\;\geq \;\lambda_{2}\;\geq \lambda_{3}$) was calculated using DTI data of *b* = 1000 s/mm^2^ and of *b* = 2000 s/mm^2^, respectively. Several diffusion indices, such as axial diffusivity (AxD or ADC), radial diffusivity (RD), mean diffusivity (MD), and fractional anisotropy (FA), were also calculated using the eigenvalues of the diffusion tensor.

### 2.5. Post-Processing of Quantitative Maps

The post-processing steps were performed using the Statistical Parametric Mapping version 12 (SPM12) software (http://www.fil.ion.ucl.ac.uk/spm/software/spm12/, Department of Imaging Neuroscience, University College, London, UK). First, the 3D T1W image and all reconstructed maps for each participant were co-registered using an affine transformation. Second, the 3D T1W images with two time points were processed using the pairwise longitudinal registration toolbox in SPM 12. The deformation maps from the longitudinal registration were then used to sample the 3D T1W image and all reconstructed maps for each time point onto the mid-point average T1 image of the respective subject. Third, the 3D T1W image was segmented into gray matter, white matter, and cerebrospinal fluid (CSF) using the computational anatomy toolbox (CAT12) tool (http://www.neuro.uni-jena.de/cat/). Fourth, the average T1 image of before and after treatment was spatially normalized into our AD-specific brain template using the CAT12 tool, and all maps were then spatially normalized into the brain template using the deformation field information from the average T1 image. Each participant’s brain tissue volume map of gray matter volume (GMV), white matter (WMV), and CSF volume was scaled by one’s total intracranial volume (TIV) value because TIV differs for each individual, especially males and females. TIV is the summation of GMV, WMV, and CSF volume. Finally, for the voxel-based statistical analysis, Gaussian smoothing was performed using a full-width at half maximum (FWHM) of 10 × 10 × 10 mm^3^ for generated and spatially normalized maps.

### 2.6. Statistical Analyses: Region-of-Interest (ROI)-Based Analyses

The main focus of this analysis was (1) to evaluate any difference in MRI measures between before and after treatment and (2) to evaluate the association of MRI measures with MMSE scores. The ROIs were defined in the brain areas of the anterior cingulate, cuneus, left and right fusiform gyrus, both hippocampus, both insula, precuneus, both thalamus, medial frontal gyrus, inferior parietal lobule, and lobes of the frontal, limbic, occipital, parietal, and temporal based on the results of the voxel-based analysis and the knowledge of the affected locations in AD patients [11]. If we can define left and right separately, then those regions were separated by the left and right sides. The atlas-based ROIs were defined using the wfu_pickatlas software (https://www.nitrc.org/projects/wfu_pickatlas, ver 3.0.5). Data extraction for each map and each participant from the selected ROIs was performed with Marsbar software ver 0.45 (Matthew Brett, https://www.nitrc.org/projects/marsbar/).

We performed the following statistical analyses using the ROI data. First, the Wilcoxon signed-rank test (paired samples) was performed to evaluate the difference in each map between before and after treatment in each group, adjusting for age difference. Adjusted *p*-values were derived via the Benjamini–Hochberg procedure across ROI-wise comparisons within each MRI index. Paired differences (Hodges–Lehmann estimates), 95% confidence intervals (CIs), and rank-biserial effect sizes were derived from paired observations of the treated participants. Second, the rank correlation test was performed to evaluate the correlation between the ROI values and all participants’ MMSE scores. For the ROI analysis, α < 0.05 was used to determine the significance level. The statistical analysis was performed using the MedCalc 22.x (https://www.medcalc.org/en/, Acacialaan, Ostend, Belgium) statistical program.

## 3. Results

### 3.1. Demographics

Table 1 lists the results of the statistical analysis of the participants’ demographic data. Age was significantly different between the control and 4 cGy groups (*p* = 0.046). Sex was not significantly different among the three groups (*p* > 0.116). MMSE scores were not significantly different among the three groups before and after LDRT. In addition, MMSE scores were not significantly different before and after LDRT in the treated groups (*p* = 0.465). To further address within-subject cognitive change over time, we performed an exploratory repeated-measures mixed-effects analysis in the treated participants (N = 6), with time as a fixed effect and subject as a random intercept. The within-subject time effect was not statistically significant (estimate, 2.000; 95% CI, [−1.042, 5.042]; *p* = 0.152).

### 3.2. ROI-Based Analyses

#### 3.2.1. Comparison Between Before and After Treatments in the LDRT-Treated Group

**Figure 1:**
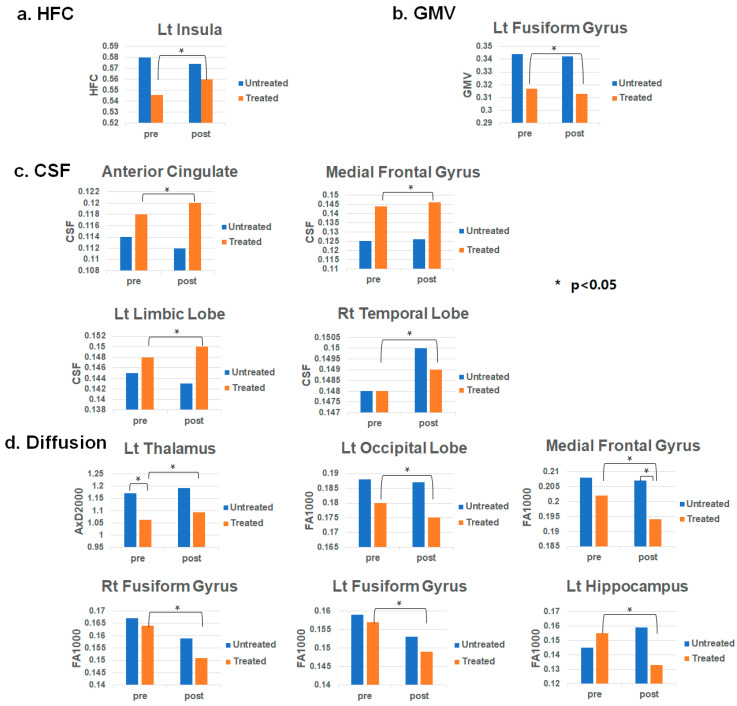
Summary of the regions-of-interest (ROI) results of MRI measures of HFC (**a**), GMV (**b**), CSF (**c**), and diffusion (**d**) of the paired comparison between before and after radiation treatment of the radiation-treated patients (N = 6) at the significant ROI areas and MRI indices. * *p* < 0.05. Abbreviations: high-frequency conductivity (HFC), brain tissue volumes of gray matter (GMV), and cerebrospinal fluid (CSF); diffusion indices of axial diffusivity (AxD), fractional anisotropy (FA).

Table 2 summarizes the results of the comparison of MRI indices between before and after the LDRT in patients (N = 6) at the significant ROI areas. Figure 1 summarizes graphically. Other entire results were listed in the Supplementary Table S1. First, HFC in the left insula was higher after LDRT than before LDRT (*p* = 0.028). Second, the CSF volumes were significantly higher after LDRT than before LDRT in the anterior cingulate (*p* = 0.028), medial frontal gyrus (*p* = 0.046), left limbic lobe (*p* = 0.046), and right temporal lobe (*p* = 0.028). GMV was significantly lower after LDRT than before LDRT in the left fusiform gyrus (*p* = 0.028). Finally, AxD from b = 2000 was markedly higher after LDRT than before LDRT. Additionally, FA was considerably lower after LDRT compared to before LDRT. However, the results of multiple comparisons were diminished in significance, as shown in the adjustment *p*-value column in Table 2 and Supplementary Table S1. Supplementary Figure S2 shows voxel-based paired t-test results comparing pre- and post-treatment maps for HFC and CSFV. The detailed locations of the significant association areas are listed in Supplementary Table S3.

#### 3.2.2. Correlation Between Each MRI Measure and MMSE Scores Before and After Treatment

Table 3 summarizes the significant ROI areas and MRI measures of the results of the rank correlation analysis with the MMSE scores from all participants (N = 9) for before and after radiation treatment. Other entire results were listed in the Supplementary Table S2. First, HFC was positively correlated with MMSE scores in the left fusiform gyrus (r = 0.843, *p* = 0.0043) after treatment. Second, GMV was positively correlated with MMSE scores in the inferior parietal lobule (r = 0.809, *p* = 0.0083) after treatment. Finally, diffusion indices had negative or positive correlations with MMSE scores, depending on the ROIs. Supplementary Figure S3 shows the exploratory voxel-based multiple regression analysis between MMSE scores and MRI metrics of HFC and FA. Post-treatment HFC showed a negative association with MMSE scores, while pre-treatment FA (at b = 1000 and 2000 s/mm^2^) was negatively associated with MMSE scores. The detailed locations of the significant association areas are listed in Supplementary Table S4.

## 4. Discussion

### 4.1. High-Frequency Conductivity

HFC represents the ionic conductivity of tissue—how easily electrical charges move in response to an electromagnetic field. This property increases with ion concentration and ion mobility in tissue. Both AD pathology and radiation-induced microenvironmental changes can influence these parameters. Conductivity was derived using MREPT, a technique that reconstructs conductivity from B_1_ field maps. Conductivity is computed at the Larmor frequency (~128 MHz at 3T) and derived from the double derivative of the B_1_ field equation (Equation (1)) and the phase-based MREPT formula (Equation (2)). Numerical solution uses a finite-difference method with regularization. Conductivity is sensitive to pathological and physiological conditions (e.g., inflammation, edema, and altered cellularity). Previous studies have demonstrated that conductivity is a sensitive MRI index for detecting high-dose radiation-induced tissue changes in mouse brain tumors [12]. LDRT is hypothesized to modulate inflammation, microglial activation, edema, and BBB permeability. These processes affect ionic homeostasis, which conductivity captures effectively. ROI-based results showed that HFC showed a strong positive correlation with cognitive improvement. HFC in the left fusiform gyrus correlated positively with MMSE scores after treatment (r = 0.843, *p* = 0.0043). This suggests that conductivity changes may reflect beneficial biological responses to LDRT.

### 4.2. Diffusivity

DTI quantifies the mobility of water molecules in tissue. Each DTI metric is sensitive to different microstructural features: For example, AxD indicates axonal integrity, RD indicates myelin changes, MD indicates overall diffusion magnitude (often increased in neurodegeneration), and FA indicates microstructural organization and fiber integrity. These metrics are sensitive to inflammation, cellularity, extracellular space changes, and demyelination—all processes potentially influenced by LDRT. Diffusion was shown to be the sensitive index for detecting radiation-induced changes (after conductivity) in mouse brain tumors [12]. ROI-based results showed significant post-LDRT differences in multiple regions. AxD2000 in the left thalamus was higher after treatment (*p* = 0.046). FA changes were observed in the fusiform gyri, hippocampus, and occipital regions (*p* < 0.05). After LDRT, DTI metrics demonstrated robust correlations with MMSE scores. DTI indices of AxD with b = 1000 and 2000, MD with b = 1000 and 2000, and RD in the fusiform gyrus were strongly negatively correlated (r from −0.758 to −0.826). In addition, many indices in the insula and thalamus positively correlated with MMSE scores. Furthermore, the limbic and occipital regions also showed significant DTI–cognition correlations (Table 3). The observed patterns suggest that LDRT may stabilize or reduce water diffusivity, potentially reflecting attenuated neuroinflammation or improved microstructural organization in regions associated with cognitive performance.

### 4.3. Importance of Current Study

Conductivity (HFC) captures ionic and electromagnetic changes that reflect tissue physiology and microenvironmental alterations from LDRT (e.g., inflammation, edema, and permeability changes). Diffusion (DTI) captures microstructural changes in extracellular and intracellular water mobility. Together, these two biomarkers may provide a multi-modal view of Inflammation and microglial activation, extracellular space modulation, axonal and myelin integrity, tissue ionic changes, and potential normalization of pathological processes. Both HFC and DTI measures changed significantly after LDRT in multiple brain regions. Both correlated with cognitive scores, indicating biological relevance. Conductivity showed the strongest association with cognitive improvement, suggesting it may be the more direct biomarker of the LDRT effect. The combined use of conductivity and diffusion offers a powerful, quantitative imaging framework to assess the biological and cognitive impact of LDRT in AD, capturing both ionic/microenvironmental and microstructural tissue responses.

Accumulating preclinical evidence indicates that LDRT can modulate multiple pathological processes implicated in AD. Across diverse animal models, LDRT has been shown to reduce amyloid burden, attenuate neuroinflammation, and preserve neuronal and synaptic integrity, collectively supporting its potential as a disease-modifying intervention rather than a purely symptomatic therapy [18,19,20,21].

One of the most consistent findings across preclinical studies is the reduction in both soluble and aggregated amyloid-β following LDRT. This effect has been observed across multiple AD models, including APP/PS1 mice, 3xTg-AD mice, and TgF344-AD rats, and across a range of radiation doses and fractionation schedules. These findings suggest that amyloid modulation by LDRT is not model-specific and may represent a generalizable biological response. Proposed mechanisms include direct destabilization of amyloid fibrils through disruption of hydrogen bonding, as well as enhanced clearance mediated by microglial activation and immune cell trafficking [6,7,8].

In parallel, LDRT consistently demonstrates immunomodulatory effects, particularly in suppressing chronic neuroinflammation. Multiple studies report reductions in microgliosis, astrogliosis, and pro-inflammatory cytokine expression following LDRT. Importantly, several investigations indicate that LDRT promotes a shift in microglial phenotype toward an M2-like, reparative state, which is associated with debris clearance and neuroprotection rather than neurotoxicity. These immunological effects appear to be dose- and schedule-dependent, with fractionated regimens in the range of 0.1–1 Gy per fraction most consistently associated with beneficial outcomes.

Beyond histopathological changes, LDRT has been shown to confer neuroprotective effects at the structural and functional levels. The preservation of hippocampal architecture, reduced synaptic loss, and improved performance in learning and memory tasks have been reported across multiple models. These functional improvements support the biological relevance of LDRT-induced molecular and cellular changes and suggest that modulation of the neuroinflammatory milieu may translate into preserved cognitive circuitry.

Importantly, the biologically effective dose window for LDRT in AD models appears to be substantially lower than that used in conventional oncologic radiotherapy. Across studies, cumulative doses typically range from approximately 3 to 10 Gy, delivered fractionally, although measurable effects have also been observed at doses below 1 Gy depending on radiation type and timing [19]. This low-dose range is associated with minimal tissue toxicity and suggests a favorable therapeutic index for non-malignant neurodegenerative conditions.

Early clinical observations, though limited in scale, are broadly consistent with preclinical findings. Case reports and small pilot studies suggest that LDRT is feasible and well tolerated in patients with AD, with no evidence of radiation-induced cognitive decline and occasional signals of cognitive stabilization or improvement [14,22]. While these studies are underpowered to establish efficacy, their safety profiles and concordance with mechanistic animal data justify further controlled clinical trials.

Preclinical and early clinical evidence support the concept that LDRT engages convergent biological mechanisms—including amyloid destabilization, modulation of the blood–brain barrier, suppression of pro-inflammatory cytokines, and microglial reprogramming—that collectively may slow or modify AD pathology. The present study builds on this foundation by incorporating quantitative neuroimaging biomarkers to objectively assess brain-level responses to LDRT, thereby strengthening the translational bridge between experimental models and human disease.

### 4.4. Study Limitation

This study has several limitations that should be considered when interpreting the findings and their generalizability. First, the study involved only six treated participants and three untreated participants and used a non-randomized and non-blinded pilot design, which substantially limits internal validity, the statistical power to detect subtle effects, and generalizability. With a small sample, effect-size estimates can be unstable and more susceptible to sampling variability, and subgroup analyses in particular may be underpowered. In particular, the lack of randomization may have introduced selection bias, and the lack of formal blinding may have affected MMSE-based cognitive assessment as well as MRI post-processing and interpretation. Therefore, the present findings should be regarded as exploratory and require confirmation in larger randomized, assessor-blinded studies. Second, optimal dose, fractionation, and patient selection are unknown. We decided those based on the results of the previous studies. Further investigation must be carried out to optimize a study trial. Third, although we evaluated conductivity and diffusivity changes after LDRF, the underlying mechanisms for why those parameters are changed after LDRT remain unclear. Therefore, animal studies are recommended to validate our outcomes. Fourth, MRI data were acquired at only one specialized institute, reducing consistency across imaging platforms. Fifth, a short follow-up duration (6 months) may not capture the delayed or long-term effects of LDRT. Sixth, MREPT reconstruction to map conductivity was sensitive to noise and required regularization, potentially affecting accuracy. Finally, an investigation should be further performed with stratified AD stages and genotypes, and the long-term effect.

## 5. Conclusions

This exploratory study provides the first clinical evidence that LDRT induces significant physiological and microstructural changes in the brains of patients with mild AD, detectable using advanced multi-modal MRI. HFC emerged as the sensitive biomarker, showing strong correlations with cognitive performance. Diffusion metrics also reflected meaningful treatment-related alterations. Although this study was exploratory and limited by a small sample size, these findings support the feasibility of using quantitative MRI to monitor LDRT responses in AD, highlighting conductivity and diffusion as promising biomarkers for future clinical trials.

## Figures and Tables

**Table 1:** Demographic characteristics of this study.

Group	Age (Years) Before RT	Sex	MMSE Score		
			Before LDRT	After LDRT	*p*-Value
Control (CT) (N = 3) (total 0 cGy)	68 (75, 67, 68)	F, M, M	19 (23, 19, 19)	24 (24, 19, 25)	N/A
4 cGy (N = 3) (total 24 cGy)	76 (76, 76, 77)	F, M, M	24 (24, 22, 24)	24 (28, 22, 24)	0.465 †
50 cGy (N = 3) (total 300 cGy)	76 (64, 78, 76)	M, M, F	22 (22, 24, 19)	22 (21, 22, 22)	
*p*-value (0 cGy vs. 4 cGy)	0.046 *	0.157 ‡	0.116 *	0.658 *	N/A
*p*-value (0 cGy vs. 50 cGy)	0.700 *	0.480 ‡	0.487 *	0.507 *	
*p*-value (4 cGy vs. 50 cGy)	0.817 *	0.480 ‡	0.346 *	0.105 *	

Age and MMSE scores are listed in median (measured value). * Mann–Whitney test, † Wilcoxon signed-rank test, ‡ Chi-squared test. Abbreviations: female (F), male (M), low-dose radiation treatment (RT).

**Table 2:** Result of paired comparison of MRI measure indices between before and after radiation treatment of the radiation-treated patients using both 4 cGy and 50 cGy (N = 6).

ROIs	MRI Indices	Before	After	Effect Size	* *p*-Value	Adj. *p*-Value
Lt Insula	HFC	0.55 (0.52 to 0.61)	0.56 (0.53 to 0.63)	1.00	0.028	0.112
AC	CSF	0.12 (0.09 to 0.16)	0.12 (0.09 to 0.16)	1.00	0.028	0.057
Lt Fusiform Gyrus	GM	0.32 (0.28 to 0.33)	0.31 (0.27 to 0.32)	−1.00	0.028	0.140
MFG	CSF	0.14 (0.11 to 0.15)	0.15 (0.11 to 0.15)	0.91	0.046	0.057
Lt Limbic Lobe	CSF	0.15 (0.10 to 0.18)	0.15 (0.10 to 0.18)	0.91	0.046	0.057
Rt Temporal Lobe	CSF	0.15 (0.10 to 0.19)	0.15 (0.11 to 0.19)	1.00	0.028	0.057
Lt Fusiform Gyru	FA1000	0.16 (0.15 to 0.20)	0.15 (0.03 to 0.16)	−1.00	0.028	0.055
Rt Fusiform Gyrus	FA1000	0.16 (0.15 to 0.18)	0.15 (0.03 to 0.17)	−0.91	0.046	0.055
Lt Hippocampus	FA1000	0.16 (0.13 to 0.18)	0.13 (0.10 to 0.17)	−0.91	0.046	0.055
Lt Thalamus	AxD2000	1.06 (1.02 to 1.10)	1.09 (1.08 to 1.20)	0.91	0.046	0.276
MFG	FA1000	0.20 (0.19 to 0.21)	0.19 (0.19 to 0.20)	−1.00	0.028	0.055
Lt Occipital Lobe	FA1000	0.18 (0.18 to 0.21)	0.18 (0.16 to 0.19)	−0.91	0.046	0.055

Data are presented as the median (95% confidence interval for the median). * *p*-values were derived from the Wilcoxon signed-rank test for paired observations. For the untreated group, we cannot estimate the *p*-value due to the too small sample size (N = 3). Adjusted *p*-values (Adj. *p*) were calculated using the Benjamini–Hochberg procedure across all ROI-wise comparisons within each specific MRI index to control for the false discovery rate. Paired differences (Hodges–Lehmann estimates), 95% confidence intervals (CIs), and rank-biserial effect sizes were derived from paired observations of the treated participants. Abbreviations: anterior cingulate (AC), medial frontal gyrus (MFG), high-frequency conductivity (HFC), and cerebrospinal fluid (CSF); diffusion indices of axial diffusivity (AxD), fractional anisotropy (FA).

**Table 3:** Summary of the significant region-of-interest (ROI) areas and MRI measures of the results of the rank correlation analysis with the Mini-Mental State Examination (MMSE) scores from all participants (N = 9) after radiation treatment.

		After Treatment				
ROI	Index	rho	*p*-Value	Adj. *p*-Value	Leave-One-Out (Range)	Sign Consistency
AC	FA2000	−0.690	0.040	0.212	−0.830 to−0.552	9/9
Lt Fusiform Gyrus	HFC	0.843	0.004	0.100	0.805 to 0.878	9/9
	AxD1000	−0.826	0.006	0.047	−0.904 to −0.749	9/9
	AxD2000	−0.766	0.016	0.092	−0.835 to −0.663	9/9
	MD1000	−0.826	0.006	0.070	−0.904 to −0.749	9/9
	MD2000	−0.758	0.018	0.138	−0.822 to −0.651	9/9
	RD1000	−0.826	0.006	0.140	−0.904 to −0.749	9/9
	RD2000	−0.826	0.006	0.140	−0.904 to −0.749	9/9
Lt Insula	AxD1000	0.766	0.016	0.092	0.663 to 0.855	9/9
	AxD2000	0.834	0.005	0.070	0.761 to 0.878	9/9
Rt Insula	AxD1000	0.826	0.006	0.047	0.749 to 0.896	9/9
	AxD2000	0.826	0.006	0.070	0.749 to 0.896	9/9
	FA1000	0.792	0.011	0.253	0.732 to 0.855	9/9
	FA2000	0.758	0.018	0.191	0.699 to 0.795	9/9
	MD1000	0.741	0.022	0.172	0.626 to 0.896	9/9
	MD2000	0.766	0.016	0.138	0.663 to 0.933	9/9
	RD1000	0.741	0.022	0.172	0.626 to 0.896	9/9
Lt Thalamus	FA1000	0.715	0.030	0.306	0.589 to 0.830	9/9
	FA2000	0.732	0.025	0.191	0.663 to 0.807	9/9
IPL	GMV	0.809	0.008	0.191	0.732 to 0.896	9/9
Rt Limbic Lobe	AxD1000	0.834	0.005	0.047	0.761 to 0.878	9/9
	AxD2000	0.766	0.016	0.092	0.663 to 0.855	9/9
	MD1000	0.851	0.004	0.070	0.786 to 0.903	9/9
Rt Occipital Lobe	FA1000	−0.690	0.040	0.306	−0.830 to −0.552	9/9
	FA2000	−0.800	0.010	0.191	−0.892 to −0.712	9/9

Spearman rank correlation coefficients (rho, ρ) are presented for the association between MMSE scores and MRI metrics (N = 9). Adj. *p*-values were calculated using the Benjamini–Hochberg procedure within each MRI index separately for the before-treatment and after-treatment analyses. Leave-one-out (LOO) sensitivity analyses were performed by iteratively excluding one participant to evaluate the stability of the correlation. LOO sign consistency indicates the frequency (out of 9 iterations) with which the direction of the correlation remained unchanged. All results are listed in the Supplementary Table S2. Abbreviation: anterior cingulate (AC), inferior parietal lobule (IPL), high-frequency conductivity (HFC), brain tissue volumes of gray matter (GMV); diffusion indices of axial diffusivity (AxD), fractional anisotropy (FA), mean diffusivity (MD), and radial diffusivity (RD) with b = 1000 and 2000.

## Data Availability

The datasets used and/or analyzed in the current study are available from the corresponding author on reasonable request.

## Supplementary Materials

The following supporting information can be downloaded at: https://www.mdpi.com/article/10.3390/diagnostics16081163/s1, design of the clinical trial, results of region-of-interest (ROI) analyses (Supplementary Table S1, Supplementary Figure S1, and Supplementary Table S2), and results of voxel-based analyses (Supplementary Figure S2, Supplementary Table S3, Supplementary Figure S3, and Supplementary Table S4).

## Abbreviations

The following abbreviations are used in this manuscript:

AD	Alzheimer’s disease
CDR	clinical dementia rating
MMSE	Mini-Mental State Examination
3D T1W	three-dimensional (3D) T1-weighted (T1W)
CSFV	cerebrospinal fluid (CSF) volume
GMV	gray matter volume
WMV	white matter volume
HFC	high-frequency conductivity
FA	fractional anisotropy
MD	mean diffusivity
RD	radial diffusivity
AxD	axial diffusivity
LDRT	low-dose radiation treatment
ROI	region-of-interest

## References

[1] Querfurth H.W., LaFerla F.M. (2010). Alzheimer’s disease. N. Engl. J. Med..

[2] Leng F., Edison P. (2021). Neuroinflammation and microglial activation in Alzheimer disease: Where do we go from here?. Nat. Rev. Neurol..

[3] Gondi V., Pugh S.L., Tome W.A., Caine C., Corn B., Kanner A., Rowley H., Kundapur V., DeNittis A., Greenspoon J.N. (2014). Preservation of memory with conformal avoidance of the hippocampal neural stem-cell compartment during whole-brain radiotherapy for brain metastases (RTOG 0933): A phase II multi-institutional trial. J. Clin. Oncol..

[4] Kurrus J.A., Hayes J.K., Hoidal J.R., Menendez M.M., Elstad M.R. (1998). Radiation therapy for tracheobronchial amyloidosis. Chest.

[5] Neben-Wittich M.A., Foote R.L., Kalra S. (2007). External beam radiation therapy for tracheobronchial amyloidosis. Chest.

[6] Bistolfi F. (2008). Localized amyloidosis and Alzheimer’s disease: The rationale for weekly long-term low dose amyloid-based fractionated radiotherapy. Neuroradiol. J..

[7] Betlazar C., Middleton R.J., Banati R.B., Liu G.J. (2016). The impact of high and low dose ionising radiation on the central nervous system. Redox Biol..

[8] Hohsfield L.A., Humpel C. (2015). Migration of blood cells to beta-amyloid plaques in Alzheimer’s disease. Exp. Gerontol..

[9] Katscher U., Voigt T., Findeklee C., Vernickel P., Nehrke K., Doessel O. (2009). Determination of electric conductivity and local SAR via B1 mapping. IEEE Trans. Med. Imaging.

[10] Katscher U., van den Berg C.A.T. (2017). Electric properties tomography: Biochemical, physical and technical background, evaluation and clinical applications. NMR Biomed..

[11] Park S., Jung S.M., Lee M.B., Rhee H.Y., Ryu C.W., Cho A.R., Kwon O.I., Jahng G.H. (2022). Application of High-Frequency Conductivity Map Using MRI to Evaluate It in the Brain of Alzheimer’s Disease Patients. Front. Neurol..

[12] Park J.A., Kim Y., Yang J., Choi B.K., Katoch N., Park S., Hur Y.H., Kim J.W., Kim H.J., Kim H.C. (2023). Effects of Irradiation on Brain Tumors Using MR-Based Electrical Conductivity Imaging. Cancers.

[13] Kim D.-Y., Kim J.S., Seo Y.-S., Park W.-Y., Kim B.H., Hong E.-H., Kim J.Y., Cho S.-J., Rhee H.Y., Kim A. (2023). Evaluation of Efficacy and Safety Using Low Dose Radiation Therapy with Alzheimer’s Disease: A Protocol for Multicenter Phase II Clinical Trial. J. Alzheimer’s Dis..

[14] Cuttler J.M., Moore E.R., Hosfeld V.D., Nadolski D.L. (2016). Treatment of Alzheimer Disease With CT Scans: A Case Report. Dose Response.

[15] Yang E.-J., Kim H., Choi Y., Kim H.J., Kim J.H., Yoon J., Seo Y.-S., Kim H.-S. (2021). Modulation of Neuroinflammation by Low-Dose Radiation Therapy in an Animal Model of Alzheimer’s Disease. Int. J. Radiat. Oncol. Biol. Phys..

[16] Voigt T., Homann H., Katscher U., Doessel O. (2012). Patient-individual local SAR determination: In vivo measurements and numerical validation. Magn. Reson. Med..

[17] Gurler N., Ider Y.Z. (2017). Gradient-based electrical conductivity imaging using MR phase. Magn. Reson. Med..

[18] Ceyzériat K., Tournier B.B., Millet P., Dipasquale G., Koutsouvelis N., Frisoni G.B., Garibotto V., Zilli T. (2022). Low-Dose Radiation Therapy Reduces Amyloid Load in Young 3xTg-AD Mice. J. Alzheimer’s Dis..

[19] Chicheva M.M., Mal’tsev A.V., Kokhan V.S., Bachurin S.O. (2020). The Effect of Ionizing Radiation on Cognitive Functions in Mouse Models of Alzheimer’s Disease. Dokl. Biol. Sci..

[20] Marples B., McGee M., Callan S., Bowen S.E., Thibodeau B.J., Michael D.B., Wilson G.D., Maddens M.E., Fontanesi J., Martinez A.A. (2016). Cranial irradiation significantly reduces beta amyloid plaques in the brain and improves cognition in a murine model of Alzheimer’s Disease (AD). Radiother. Oncol..

[21] Wilson G.D., Rogers C.L., Mehta M.P., Marples B., Michael D.B., Welsh J.S., Martinez A.A., Fontanesi J. (2023). The Rationale for Radiation Therapy in Alzheimer’s Disease. Radiat. Res..

[22] Cuttler J.M., Abdellah E., Goldberg Y., Al-Shamaa S., Symons S.P., Black S.E., Freedman M. (2021). Low Doses of Ionizing Radiation as a Treatment for Alzheimer’s Disease: A Pilot Study. J. Alzheimer’s Dis..

