# Supplementary material for: Evaluation of Low-Dose Radiation Treatment Effects Using Conductivity, Diffusivity, and Brain Tissue Volumes Treated in Patients with Mild Alzheimer’s Disease: Exploratory Investigation
Source: Diagnostics (Basel). 2026 Apr 14;16(8):1163. doi: 10.3390/diagnostics16081163 (PMC13115071; doi:10.3390/diagnostics16081163)
Supplement: Supplementary file 1 [file diagnostics-16-01163-s001.zip › diagnostics-4222568-supplementary.pdf]

Evaluation of low-dose radiation treatment effects using conductivity, diffusivity, and brain tissue volumes treated in patients with mild Alzheimer's disease: Exploratory Investigation

Supplementary Materials

1. Design of the Clinical Trial

The present investigation was designed based on a previously published multicenter clinical trial protocol evaluating the efficacy and safety of low-dose radiation therapy (LDRT) in patients with Alzheimer's disease (Kim et al., 2023). This trial was registered at ClinicalTrials.gov (Identifier: NCT05635968) and conducted as a multicenter, controlled, open-label study aimed at evaluating the efficacy and safety of low-dose whole-brain radiotherapy in patients with mild to moderate Alzheimer's disease.

After providing written informed consent, participants underwent screening evaluations including cognitive assessments, brain magnetic resonance imaging (MRI), and amyloid PET scans to determine eligibility based on predefined inclusion and exclusion criteria. Eligible participants were subsequently assigned a randomization number.

Participants in the experimental groups received whole-brain radiotherapy according to predefined radiation dose schedules, delivered in six fractions over 3 weeks. The control group received sham (placebo) radiotherapy following the same treatment schedule. Follow-up visits for efficacy and safety assessments were conducted approximately 1 month, 6 months, and 12 months after completion of radiotherapy.

Recruitment of Study Participants: Participants were recruited from patients with Alzheimer's disease visiting the neurology departments of participating institutions, based on referrals from treating neurologists. Additionally, recruitment notices were posted both within and outside the institutions to facilitate enrollment. No potential participants were excluded based on race or socioeconomic status.

Randomization and Allocation: A total of 36 patients with mild to moderate Alzheimer 's-type dementia were enrolled and randomly assigned to three groups using stratified block randomization. Stratification factors included neuropsychological test scores, age, physical performance, and amyloid PET standardized uptake value ratio (SUVR) to minimize imbalance among treatment groups. The random allocation sequence was generated and managed by an independent third party not involved in the study. After confirmation of eligibility and informed consent, participants were assigned screening numbers followed by randomization numbers according to the pre-generated allocation table. Group assignments were implemented using sealed opaque envelopes opened sequentially by an independent envelope manager. The study comprised three groups—two experimental groups and one control group—with 12 participants allocated to each group.

Concomitant Treatment: Standard treatments for pre-existing medical conditions were maintained throughout the study period. All concomitant medications deemed medically necessary were permitted and recorded in detail by the clinical investigators. No medications were prohibited during the study.

Cognitive Function Assessment: Cognitive assessments were conducted at baseline (screening visit), and at 28 weeks and 52 weeks after treatment initiation. The Korea-Mini-Mental State Examination (K-MMSE) and Clinical Dementia Rating (CDR) scale were used to assess cognitive and social functioning through interviews with participants and their caregivers. Additional assessments included the Alzheimer's Disease Assessment Scale–Korea (ADAS-K), Caregiver-Administered Neuropsychiatric Inventory (CGA-NPI), and the Korean Instrumental Activities of Daily Living (K-iADL).

Radiation Dose and Treatment Duration: The radiation dose and fractionation schedule were determined with reference to preclinical studies in Alzheimer's disease animal models and previously published clinical protocols (Kim et al., 2023). To minimize potential adverse effects and to differentiate this protocol from existing studies, reduced radiation doses and treatment frequency were adopted.

2. Results of Region-of-interest (ROI) Analyses

Supplementary Table S1. Result of comparison of MRI measure indices between before and after radiation treatment of the radiation-treated patients (N=6)

ROIs	Conductivity	Before	After	Effect size	*p-value	Adj.p-value
AC	HFC	0.63 (0.57 to 0.71)	0.62 (0.60 to 0.70)	-0.33	0.463	0.600
Rt Hippocampus	HFC	0.64 (0.58 to 0.72)	0.65 (0.57 to 0.75)	0.81	0.075	0.150
Lt Insula	*HFC*	*0.55 (0.52 to 0.61)*	*0.56 (0.53 to 0.63)*	*1.00*	*0.028*	*0.112*
Lt Frontal Lobe	HFC	0.62 (0.59 to 0.67)	0.61 (0.59 to 0.69)	0.24	0.6	0.600
ROIs	BTV Indices	Before	After	Effect size	*p-value	Adj.p-value
AC	*CSF*	*0.12 (0.09 to 0.16)*	*0.12 (0.09 to 0.16)*	*1.00*	*0.028*	0.057
	GM	0.29 (0.26 to 0.32)	0.29 (0.26 to 0.31)	-0.43	0.345	0.431
	WM	0.26 (0.20 to 0.32)	0.26 (0.20 to 0.32)	-0.71	0.116	0.580
Lt Fusiform Gyrus	CSF	0.14 (0.10 to 0.16)	0.14 (0.11 to 0.16)	0.43	0.345	0.345
	GM	*0.32 (0.28 to 0.33)*	*0.31 (0.27 to 0.32)*	*-1.00*	*0.028*	*0.140*
	WM	0.15 (0.12 to 0.19)	0.15 (0.12 to 0.19)	0.05	0.917	0.917
MFG	*CSF*	*0.14 (0.11 to 0.15)*	*0.15 (0.11 to 0.15)*	*0.91*	*0.046*	*0.057*
	GM	0.26 (0.22 to 0.28)	0.26 (0.22 to 0.28)	-0.52	0.249	0.415
	WM	0.26 (0.20 to 0.29)	0.26 (0.20 to 0.29)	0.05	0.917	0.917
Lt Limbic Lobe	*CSF*	*0.15 (0.10 to 0.18)*	*0.15 (0.10 to 0.18)*	*0.91*	*0.046*	*0.057*
	GM	0.29 (0.26 to 0.33)	0.29 (0.26 to 0.32)	-0.71	0.116	0.290
	WM	0.21 (0.17 to 0.25)	0.21 (0.17 to 0.25)	-0.24	0.6	0.917
Rt Temporal Lobe	*CSF*	*0.15 (0.10 to 0.19)*	*0.15 (0.11 to 0.19)*	*1.00*	*0.028*	*0.057*
	GM	0.25 (0.24 to 0.27)	0.25 (0.24 to 0.27)	-0.24	0.6	0.600
	WM	0.23 (0.20 to 0.27)	0.23 (0.20 to 0.27)	-0.24	0.6	0.917
ROIs	Diffusion Indices	Before	After	Effect size	*p-value	Adj.p-value
Lt Fusiform Gyrus	AxD1000	1.20 (1.13 to 1.26)	1.20 (0.22 to 1.30)	0.14	0.753	0.904
	AxD2000	0.99 (0.94 to 1.00)	0.99 (0.19 to 1.02)	-0.05	0.917	0.917
	FA1000	*0.16 (0.15 to 0.20)*	*0.15 (0.03 to 0.16)*	*-1.00*	*0.028*	*0.055*
	FA2000	0.14 (0.13 to 0.15)	0.14 (0.02 to 0.15)	-0.52	0.249	0.690
	MD1000	1.06 (0.97 to 1.11)	1.06 (0.20 to 1.16)	0.24	0.6	0.904
	MD2000	0.87 (0.82 to 0.89)	0.87 (0.16 to 0.92)	0.05	0.917	0.917
	RD1000	0.99 (0.89 to 1.04)	1.00 (0.18 to 1.09)	0.24	0.6	0.900
	RD2000	0.82 (0.76 to 0.84)	0.82 (0.15 to 0.86)	0.24	0.6	0.917
Rt Fusiform Gyrus	AxD1000	1.17 (1.08 to 1.28)	1.16 (0.24 to 1.20)	-0.33	0.463	0.900
	AxD2000	0.95 (0.91 to 1.04)	0.95 (0.19 to 0.99)	-0.05	0.917	0.917
	FA1000	*0.16 (0.15 to 0.18)*	*0.15 (0.03 to 0.17)*	*-0.91*	*0.046*	*0.055*
	FA2000	0.15 (0.13 to 0.15)	0.14 (0.03 to 0.16)	-0.24	0.6	0.720
	MD1000	1.02 (0.94 to 1.13)	1.03 (0.21 to 1.07)	-0.14	0.753	0.904
	MD2000	0.84 (0.80 to 0.92)	0.85 (0.17 to 0.87)	-0.14	0.753	0.917

	RD1000	0.94 (0.87 to 1.06)	0.96 (0.19 to 1.00)	-0.05	0.917	0.917
	RD2000	0.78 (0.74 to 0.86)	0.79 (0.16 to 0.81)	-0.14	0.753	0.917
Lt Hippocampus	AxD1000	1.63 (1.22 to 1.74)	1.63 (0.96 to 1.72)	-0.24	0.6	0.900
	AxD2000	1.26 (1.02 to 1.36)	1.27 (0.73 to 1.32)	-0.14	0.753	0.917
	FA1000	*0.16 (0.13 to 0.18)*	*0.13 (0.10 to 0.17)*	*-0.91*	*0.046*	*0.055*
	FA2000	0.13 (0.10 to 0.14)	0.12 (0.09 to 0.14)	-0.24	0.6	0.720
	MD1000	1.43 (1.04 to 1.51)	1.44 (0.84 to 1.52)	0.05	0.917	0.917
	MD2000	1.13 (0.90 to 1.20)	1.13 (0.64 to 1.17)	-0.33	0.463	0.917
	RD1000	1.33 (0.94 to 1.40)	1.34 (0.78 to 1.42)	0.14	0.753	0.904
	RD2000	1.06 (0.83 to 1.12)	1.06 (0.60 to 1.10)	-0.33	0.463	0.917
Lt Thalamus	AxD1000	1.38 (1.25 to 1.42)	1.41 (1.37 to 1.69)	0.71	0.116	0.696
	AxD2000	*1.06 (1.02 to 1.10)*	*1.09 (1.08 to 1.20)*	*0.91*	*0.046*	*0.276*
	FA1000	0.31 (0.28 to 0.33)	0.31 (0.28 to 0.36)	-0.14	0.753	0.753
	FA2000	0.26 (0.26 to 0.28)	0.27 (0.25 to 0.33)	0.14	0.753	0.753
	MD1000	1.06 (0.99 to 1.12)	1.11 (1.09 to 1.31)	0.81	0.075	0.450
	MD2000	0.85 (0.81 to 0.89)	0.88 (0.85 to 0.93)	0.71	0.116	0.696
	RD1000	0.90 (0.85 to 0.97)	0.97 (0.93 to 1.12)	0.81	0.075	0.450
	RD2000	0.74 (0.70 to 0.78)	0.77 (0.74 to 0.81)	0.71	0.116	0.696
MFG	AxD1000	1.25 (1.16 to 1.36)	1.27 (1.16 to 1.32)	-0.33	0.463	0.900
	AxD2000	1.02 (0.96 to 1.08)	1.03 (0.94 to 1.08)	-0.24	0.6	0.917
	FA1000	*0.20 (0.19 to 0.21)*	*0.19 (0.19 to 0.20)*	*-1.00*	*0.028*	*0.055*
	FA2000	0.18 (0.17 to 0.19)	0.18 (0.17 to 0.18)	-0.43	0.345	0.690
	MD1000	1.08 (1.00 to 1.17)	1.09 (0.98 to 1.15)	-0.33	0.463	0.904
	MD2000	0.88 (0.82 to 0.94)	0.89 (0.79 to 0.93)	-0.05	0.917	0.917
	RD1000	0.99 (0.91 to 1.08)	1.01 (0.89 to 1.06)	-0.24	0.6	0.900
	RD2000	0.81 (0.76 to 0.87)	0.82 (0.71 to 0.86)	0.05	0.917	0.917
Lt Occipital Lobe	AxD1000	1.23 (1.21 to 1.27)	1.25 (0.97 to 1.26)	0.05	0.917	0.917
	AxD2000	1.00 (0.96 to 1.02)	1.00 (0.76 to 1.02)	-0.14	0.753	0.917
	FA1000	*0.18 (0.18 to 0.21)*	*0.18 (0.16 to 0.19)*	*-0.91*	*0.046*	*0.055*
	FA2000	0.17 (0.16 to 0.17)	0.16 (0.13 to 0.17)	-0.71	0.116	0.690
	MD1000	1.07 (1.04 to 1.11)	1.09 (0.83 to 1.11)	0.14	0.753	0.904
	MD2000	0.87 (0.83 to 0.88)	0.87 (0.66 to 0.89)	0.05	0.917	0.917
	RD1000	0.99 (0.95 to 1.03)	1.01 (0.76 to 1.03)	0.24	0.6	0.900
	RD2000	0.80 (0.77 to 0.82)	0.80 (0.61 to 0.83)	0.05	0.917	0.917

Data are presented as the median (95% confidence interval for the median). *p-values were derived from the Wilcoxon signed-rank test for paired observations. For the untreated group, we cannot estimate the P-value due to the too small sample size (N=3). Adjusted p-values (Adj. p) were calculated using the Benjamini-Hochberg procedure across all ROI-wise comparisons within each specific MRI index to control for the false discovery rate. Paired differences (Hodges–Lehmann estimates), 95% confidence intervals (CIs), and rank-biserial effect sizes were derived from paired observations of the treated participants. Abbreviation: anterior cingulate (AC), medial frontal gyrus (MFG), high-frequency conductivity (HFC); brain tissue volumes (BTV) of gray matter (GM), white matter (WM), and cerebrospinal fluid (CSF); diffusion indices of axial diffusivity (AxD), fractional anisotropy (FA), mean diffusivity (MD), and radial diffusivity (RD) with b=1000 and 2000.

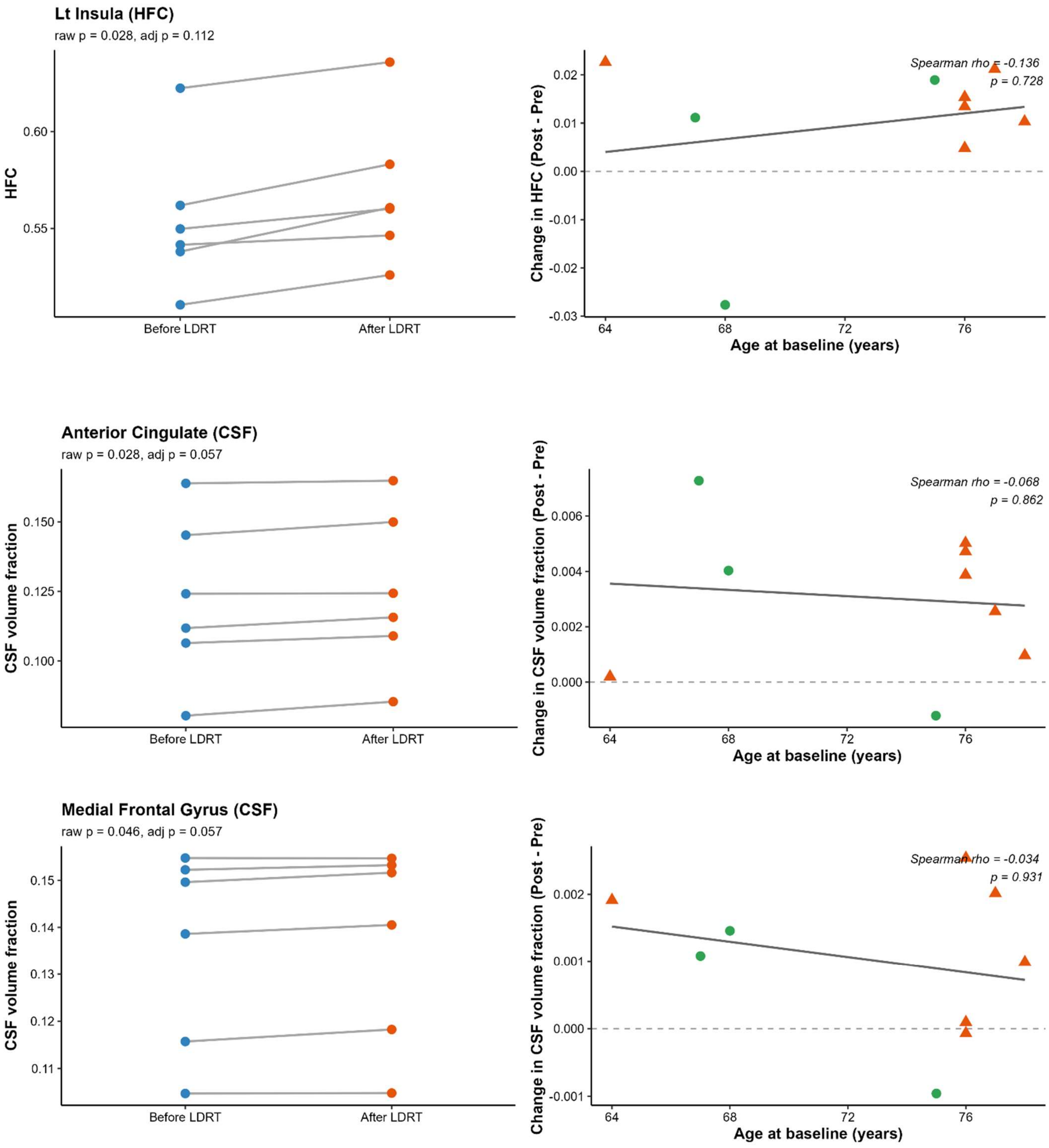

Lt Insula (HFC)
raw p = 0.028, adj p = 0.112
HFC
0.60
0.55
Before LDRT
After LDRT
Spearman rho = -0.136
p = 0.728
Change in HFC (Post - Pre)
0.02
0.01
0.00
-0.01
-0.02
-0.03
64
68
72
76
Age at baseline (years)
Anterior Cingulate (CSF)
raw p = 0.028, adj p = 0.057
CSF volume fraction
0.150
0.125
0.100
Before LDRT
After LDRT
Spearman rho = -0.068
p = 0.862
Change in CSF volume fraction (Post - Pre)
0.006
0.004
0.002
0.000
64
68
72
76
Age at baseline (years)
Medial Frontal Gyrus (CSF)
raw p = 0.046, adj p = 0.057
CSF volume fraction
0.15
0.14
0.13
0.12
0.11
Before LDRT
After LDRT
Spearman rho = -0.034
p = 0.931
Change in CSF volume fraction (Post - Pre)
0.002
0.001
0.000
-0.001
64
68
72
76
Age at baseline (years)

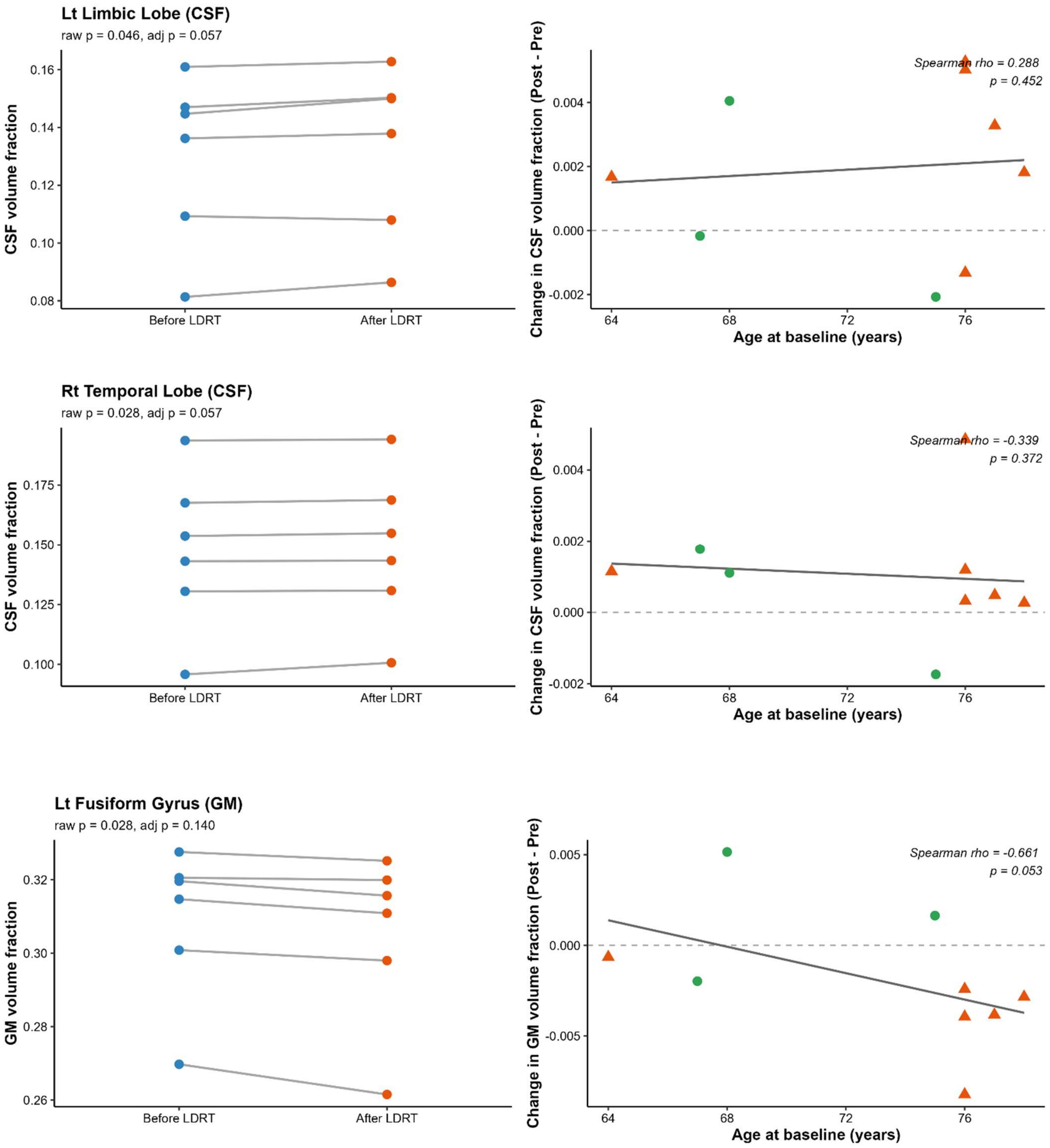
Lt Limbic Lobe (CSF)
raw p = 0.046, adj p = 0.057
CSF volume fraction
0.16
0.14
0.12
0.10
0.08
Before LDRT
After LDRT
Change in CSF volume fraction (Post - Pre)
Spearman rho = 0.288
p = 0.452
0.004
0.002
0.000
-0.002
64
68
72
76
Age at baseline (years)
Rt Temporal Lobe (CSF)
raw p = 0.028, adj p = 0.057
CSF volume fraction
0.175
0.150
0.125
0.100
Before LDRT
After LDRT
Change in CSF volume fraction (Post - Pre)
Spearman rho = -0.339
p = 0.372
0.004
0.002
0.000
-0.002
64
68
72
76
Age at baseline (years)
Lt Fusiform Gyrus (GM)
raw p = 0.028, adj p = 0.140
GM volume fraction
0.32
0.30
0.28
0.26
Before LDRT
After LDRT
Change in GM volume fraction (Post - Pre)
Spearman rho = -0.661
p = 0.053
0.005
0.000
-0.005
64
68
72
76
Age at baseline (years)

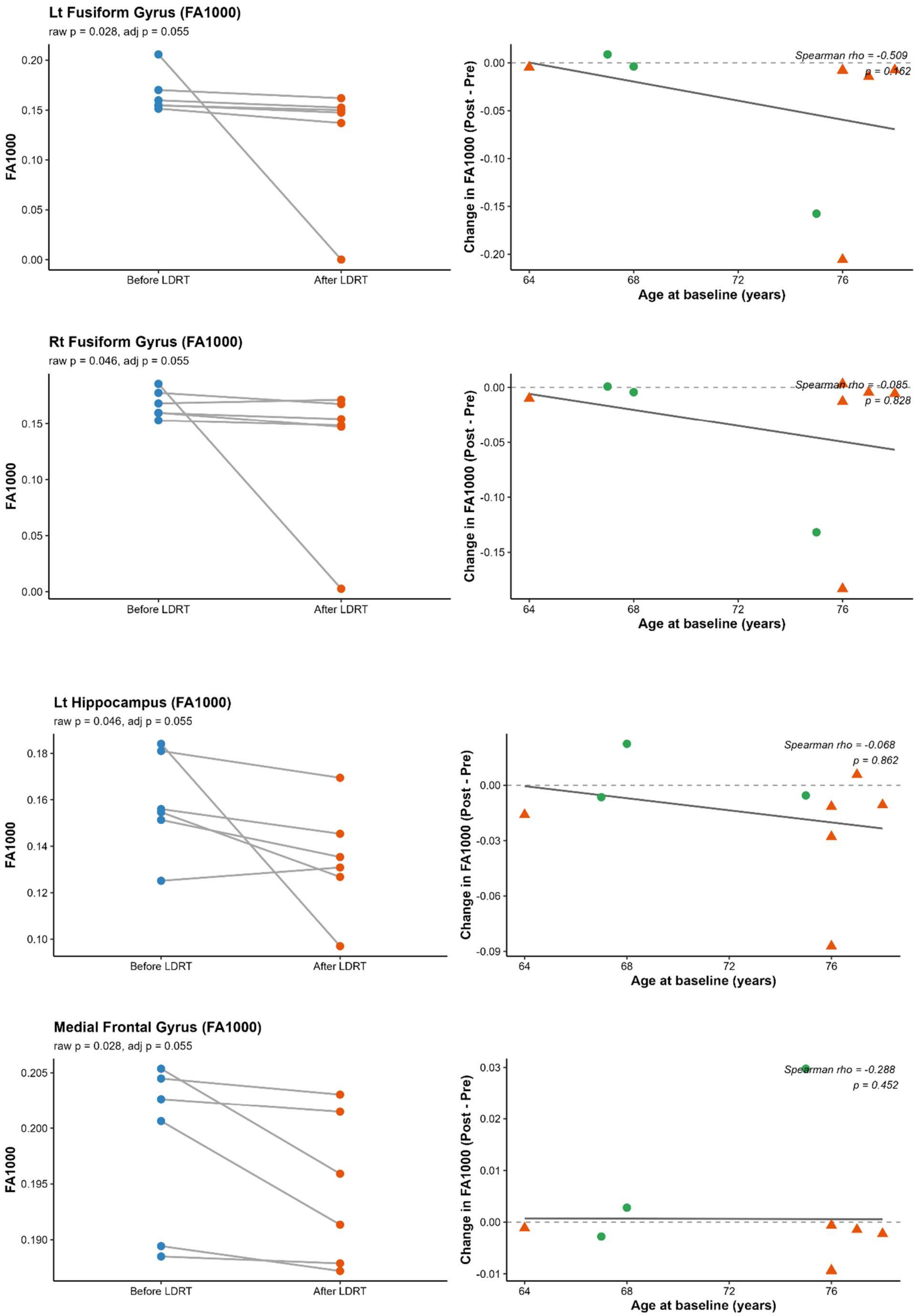
Lt Fusiform Gyrus (FA1000)
raw p = 0.028, adj p = 0.055
FA1000
Before LDRT
After LDRT
Change in FA1000 (Post - Pre)
Spearman rho = -0.509
p = 0.162
Age at baseline (years)
Rt Fusiform Gyrus (FA1000)
raw p = 0.046, adj p = 0.055
FA1000
Before LDRT
After LDRT
Change in FA1000 (Post - Pre)
Spearman rho = -0.085
p = 0.828
Age at baseline (years)
Lt Hippocampus (FA1000)
raw p = 0.046, adj p = 0.055
FA1000
Before LDRT
After LDRT
Change in FA1000 (Post - Pre)
Spearman rho = -0.068
p = 0.862
Age at baseline (years)
Medial Frontal Gyrus (FA1000)
raw p = 0.028, adj p = 0.055
FA1000
Before LDRT
After LDRT
Change in FA1000 (Post - Pre)
Spearman rho = -0.288
p = 0.452
Age at baseline (years)

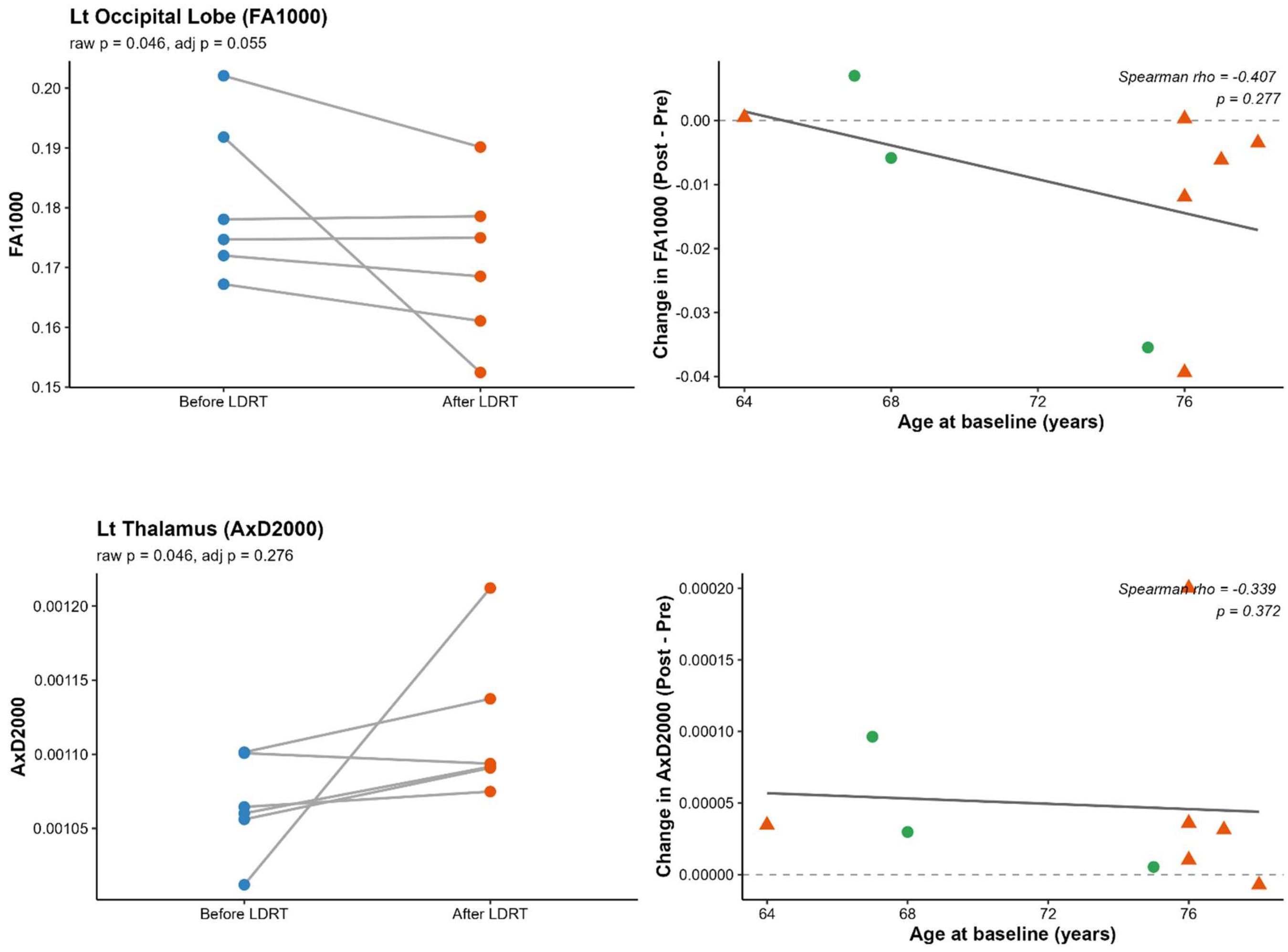

Supplementary Figure S1. Intra-subject changes in MRI metrics following LDRT and their relationship with baseline age across significant ROIs.

(Left) Paired line plots showing individual trajectories from baseline to 6 months post-LDRT (N=6 treated participants). (Right) Scatterplots showing the absolute change (Post - Pre) relative to age at baseline for the treated (orange triangles) and control (green circles) groups. The solid grey line represents the linear regression trend across the entire cohort (N=9). Spearman rho and p-values indicate that no significant age-dependent bias was observed for these changes. Abbreviation: anterior cingulate (AC), medial frontal gyrus (MFG), inferior parietal lobule (IPL), high-frequency conductivity (HFC); brain tissue volumes (BTV) of gray matter (GM), white matter (WM), and cerebrospinal fluid (CSF); diffusion indices of axial diffusivity (AxD), fractional anisotropy (FA), mean diffusivity (MD), and radial diffusivity (RD) with b=1000 and 2000.

Supplementary Table S2. Summary of all ROI areas and MRI measures of the results of the rank correlation analysis between the MMSE scores from all participants (N=9) for before and after radiation treatment.

		Before treatment					After treatment				
ROI	Index	rho	p-value	Adj.p-value	Leave-one-out(range)	Sign consistency	rho	p-value	Adj.p-value	Leave-one-out(range)	Sign consistency
AC	HFC	0.182	0.639	0.965	-0.049 to 0.544	8/9	0.068	0.862	0.862	-0.147 to 0.540	6/9
	CSF	0.130	0.739	0.982	-0.148 to 0.445	7/9	0.264	0.493	1.000	0.012 to 0.405	9/9
	GM	-0.069	0.859	0.965	-0.284 to 0.222	6/9	-0.306	0.423	0.870	-0.454 to 0.000	7/9
	WM	0.598	0.089	0.965	0.482 to 0.754	9/9	0.051	0.896	0.965	-0.135 to 0.331	5/9
	AxD1000	-0.234	0.545	0.828	-0.420 to 0.000	8/9	0.443	0.233	0.559	0.307 to 0.683	9/9
	AxD2000	-0.199	0.607	0.915	-0.420 to 0.049	8/9	0.068	0.862	0.931	-0.135 to 0.479	7/9
	FA1000	0.208	0.591	0.817	0.025 to 0.581	9/9	-0.477	0.194	0.559	-0.634 to -0.246	9/9
	FA2000	0.338	0.374	0.972	0.210 to 0.766	9/9	-0.690	0.040	0.212	-0.830 to -0.552	9/9
	MD1000	-0.156	0.689	0.869	-0.358 to 0.099	7/9	0.579	0.102	0.393	0.479 to 0.854	9/9
	MD2000	-0.251	0.514	0.835	-0.494 to -0.025	9/9	0.409	0.275	0.598	0.282 to 0.659	9/9
	RD1000	-0.156	0.689	0.869	-0.358 to 0.099	7/9	0.596	0.090	0.471	0.503 to 0.878	9/9
	RD2000	-0.217	0.576	0.909	-0.457 to 0.025	7/9	0.579	0.102	0.471	0.479 to 0.854	9/9
Cuneus	HFC	0.321	0.400	0.965	0.099 to 0.457	9/9	-0.119	0.760	0.862	-0.479 to 0.049	6/9
	CSF	0.659	0.054	0.619	0.544 to 0.851	9/9	0.264	0.493	1.000	-0.061 to 0.393	8/9
	GM	0.069	0.859	0.965	-0.235 to 0.284	7/9	0.536	0.137	0.524	0.393 to 0.712	9/9
	WM	0.355	0.348	0.965	0.161 to 0.630	9/9	0.179	0.645	0.965	-0.012 to 0.602	8/9
	AxD1000	0.659	0.054	0.412	0.544 to 0.776	9/9	0.060	0.879	0.948	-0.356 to 0.331	6/9
	AxD2000	0.555	0.121	0.915	0.408 to 0.717	9/9	-0.196	0.614	0.825	-0.528 to 0.160	8/9
	FA1000	-0.702	0.035	0.807	-0.945 to -0.593	9/9	0.043	0.913	0.955	-0.319 to 0.196	6/9
	FA2000	-0.468	0.204	0.972	-0.667 to -0.272	9/9	-0.264	0.493	0.778	-0.512 to -0.061	9/9
	MD1000	0.589	0.095	0.619	0.457 to 0.756	9/9	-0.026	0.948	0.983	-0.479 to 0.209	3/9
	MD2000	0.589	0.095	0.619	0.457 to 0.756	9/9	-0.162	0.678	0.820	-0.479 to 0.209	7/9
	RD1000	0.589	0.095	0.546	0.457 to 0.756	9/9	-0.026	0.948	0.965	-0.479 to 0.209	3/9
	RD2000	0.520	0.151	0.819	0.371 to 0.756	9/9	0.017	0.965	0.965	-0.417 to 0.270	5/9
Lt Fusiform Gyrus	HFC	0.321	0.400	0.965	0.111 to 0.531	9/9	0.843	0.004	0.100	0.805 to 0.878	9/9
	CSF	-0.009	0.982	0.982	-0.334 to 0.259	4/9	-0.426	0.253	1.000	-0.602 to -0.196	9/9
	GM	-0.269	0.485	0.965	-0.642 to -0.049	9/9	0.085	0.828	0.983	-0.270 to 0.393	7/9
	WM	0.052	0.894	0.965	-0.222 to 0.371	5/9	0.111	0.777	0.965	-0.086 to 0.602	6/9
	AxD1000	0.173	0.656	0.828	-0.062 to 0.531	8/9	-0.826	0.006	0.047	-0.904 to -0.749	9/9
	AxD2000	0.043	0.912	0.953	-0.235 to 0.358	5/9	-0.766	0.016	0.092	-0.835 to -0.663	9/9
	FA1000	0.416	0.266	0.817	0.235 to 0.729	9/9	-0.451	0.223	0.570	-0.798 to -0.209	9/9
	FA2000	0.260	0.499	0.972	0.012 to 0.544	9/9	-0.443	0.233	0.570	-0.749 to -0.196	9/9
	MD1000	0.069	0.859	0.941	-0.198 to 0.395	5/9	-0.826	0.006	0.070	-0.904 to -0.749	9/9
	MD2000	0.043	0.912	0.982	-0.235 to 0.358	5/9	-0.758	0.018	0.138	-0.822 to -0.651	9/9
	RD1000	0.035	0.929	0.929	-0.247 to 0.346	5/9	-0.826	0.006	0.140	-0.904 to -0.749	9/9
	RD2000	0.043	0.912	0.999	-0.235 to 0.358	5/9	-0.826	0.006	0.140	-0.904 to -0.749	9/9
Rt Fusiform Gyrus	HFC	0.078	0.842	0.965	-0.063 to 0.346	6/9	0.451	0.223	0.862	0.356 to 0.651	9/9
	CSF	0.633	0.068	0.619	0.519 to 0.791	9/9	-0.017	0.965	1.000	-0.233 to 0.233	6/9

	GM	-0.381	0.311	0.965	-0.556 to -0.185	9/9	0.026	0.948	0.983	-0.356 to 0.307	7/9
	WM	-0.147	0.705	0.965	-0.469 to 0.124	7/9	0.111	0.777	0.965	-0.086 to 0.602	7/9
	AxD1000	0.685	0.042	0.412	0.593 to 0.751	9/9	-0.451	0.223	0.559	-0.659 to -0.209	9/9
	AxD2000	0.728	0.026	0.525	0.642 to 0.801	9/9	-0.485	0.186	0.533	-0.708 to -0.258	9/9
	FA1000	0.130	0.739	0.850	-0.086 to 0.507	7/9	-0.528	0.144	0.474	-0.761 to -0.319	9/9
	FA2000	0.095	0.807	0.972	-0.210 to 0.296	7/9	-0.255	0.507	0.778	-0.479 to 0.074	8/9
	MD1000	0.685	0.042	0.525	0.593 to 0.751	9/9	-0.536	0.137	0.447	-0.781 to -0.331	9/9
	MD2000	0.685	0.042	0.525	0.593 to 0.751	9/9	-0.485	0.186	0.533	-0.708 to -0.258	9/9
	RD1000	0.685	0.042	0.518	0.593 to 0.751	9/9	-0.536	0.137	0.490	-0.781 to -0.331	9/9
	RD2000	0.685	0.042	0.669	0.593 to 0.751	9/9	-0.502	0.168	0.484	-0.732 to -0.282	9/9
Lt Hippocampus	HFC	-0.355	0.348	0.965	-0.630 to -0.148	9/9	-0.272	0.478	0.862	-0.439 to 0.049	7/9
	CSF	-0.043	0.912	0.982	-0.235 to 0.247	6/9	-0.562	0.115	1.000	-0.687 to -0.368	9/9
	GM	-0.208	0.591	0.965	-0.605 to -0.012	9/9	0.383	0.309	0.789	0.110 to 0.586	9/9
	WM	-0.121	0.756	0.965	-0.334 to 0.173	7/9	0.094	0.811	0.965	-0.073 to 0.442	6/9
	AxD1000	-0.139	0.722	0.828	-0.371 to 0.136	7/9	-0.341	0.370	0.733	-0.561 to -0.049	9/9
	AxD2000	-0.156	0.689	0.915	-0.408 to 0.124	7/9	-0.596	0.090	0.416	-0.723 to -0.417	9/9
	FA1000	0.199	0.607	0.817	-0.025 to 0.568	8/9	-0.128	0.743	0.955	-0.295 to 0.258	7/9
	FA2000	-0.009	0.982	0.982	-0.222 to 0.334	7/9	-0.196	0.614	0.784	-0.528 to 0.160	8/9
	MD1000	-0.199	0.607	0.869	-0.457 to 0.062	7/9	-0.383	0.309	0.638	-0.610 to -0.110	9/9
	MD2000	-0.095	0.807	0.982	-0.321 to 0.198	7/9	-0.587	0.096	0.533	-0.723 to -0.405	9/9
	RD1000	-0.165	0.672	0.869	-0.408 to 0.099	7/9	-0.383	0.309	0.588	-0.610 to -0.110	9/9
	RD2000	-0.104	0.790	0.909	-0.321 to 0.173	7/9	-0.638	0.064	0.471	-0.783 to -0.479	9/9
Rt Hippocampus	HFC	-0.355	0.348	0.965	-0.642 to -0.136	9/9	-0.366	0.333	0.862	-0.602 to -0.086	9/9
	CSF	-0.078	0.842	0.982	-0.395 to 0.062	5/9	0.051	0.896	1.000	-0.160 to 0.393	6/9
	GM	-0.260	0.499	0.965	-0.679 to -0.148	9/9	0.009	0.983	0.983	-0.430 to 0.184	6/9
	WM	-0.520	0.151	0.965	-0.741 to -0.346	9/9	0.034	0.931	0.965	-0.135 to 0.491	5/9
	AxD1000	0.000	1.000	1.000	-0.296 to 0.161	0/9	-0.281	0.464	0.733	-0.488 to 0.037	8/9
	AxD2000	0.147	0.705	0.915	-0.124 to 0.383	7/9	-0.238	0.537	0.825	-0.464 to 0.098	8/9
	FA1000	-0.503	0.168	0.817	-0.676 to -0.358	9/9	-0.162	0.678	0.955	-0.479 to 0.209	8/9
	FA2000	-0.234	0.545	0.972	-0.519 to 0.049	8/9	-0.162	0.678	0.820	-0.344 to 0.209	7/9
	MD1000	0.000	1.000	1.000	-0.296 to 0.161	0/9	-0.315	0.409	0.724	-0.537 to -0.012	9/9
	MD2000	0.069	0.859	0.982	-0.210 to 0.259	7/9	-0.400	0.286	0.598	-0.659 to -0.135	9/9
	RD1000	0.035	0.929	0.929	-0.259 to 0.210	7/9	-0.315	0.409	0.627	-0.537 to -0.012	9/9
	RD2000	0.000	1.000	1.000	-0.296 to 0.161	0/9	-0.417	0.264	0.575	-0.683 to -0.160	9/9
Lt Insula	HFC	-0.477	0.195	0.965	-0.626 to -0.284	9/9	-0.230	0.552	0.862	-0.410 to -0.012	9/9
	CSF	0.139	0.722	0.982	-0.136 to 0.457	7/9	0.323	0.396	1.000	0.098 to 0.589	9/9
	GM	0.017	0.965	0.965	-0.309 to 0.284	7/9	0.264	0.493	0.870	0.000 to 0.491	8/9
	WM	-0.113	0.773	0.965	-0.469 to 0.124	6/9	-0.332	0.383	0.965	-0.491 to -0.037	9/9
	AxD1000	0.208	0.591	0.828	-0.012 to 0.494	8/9	0.766	0.016	0.092	0.663 to 0.855	9/9
	AxD2000	0.208	0.591	0.915	0.000 to 0.593	8/9	0.834	0.005	0.070	0.761 to 0.878	9/9
	FA1000	-0.104	0.790	0.865	-0.252 to 0.099	7/9	0.545	0.129	0.474	0.405 to 0.805	9/9
	FA2000	0.052	0.894	0.972	-0.247 to 0.247	6/9	0.494	0.177	0.570	0.270 to 0.675	9/9
	MD1000	0.182	0.639	0.869	-0.049 to 0.544	8/9	0.621	0.074	0.393	0.454 to 0.771	9/9

	MD2000	0.052	0.894	0.982	-0.247 to 0.247	6/9	0.494	0.177	0.533	0.270 to 0.675	9/9
	RD1000	0.156	0.689	0.869	-0.086 to 0.507	8/9	0.494	0.177	0.490	0.270 to 0.663	9/9
	RD2000	0.130	0.739	0.909	-0.148 to 0.346	7/9	0.434	0.243	0.575	0.184 to 0.602	9/9
Rt Insula	HFC	-0.052	0.894	0.965	-0.358 to 0.235	5/9	0.179	0.645	0.862	-0.184 to 0.503	8/9
	CSF	0.182	0.639	0.982	-0.074 to 0.519	7/9	0.664	0.051	1.000	0.589 to 0.807	9/9
	GM	-0.217	0.576	0.965	-0.482 to 0.037	8/9	0.060	0.879	0.983	-0.160 to 0.528	6/9
	WM	-0.069	0.859	0.965	-0.420 to 0.124	6/9	-0.255	0.507	0.965	-0.442 to 0.049	7/9
	AxD1000	0.520	0.151	0.773	0.395 to 0.766	9/9	0.826	0.006	0.047	0.749 to 0.896	9/9
	AxD2000	0.373	0.323	0.915	0.259 to 0.717	9/9	0.826	0.006	0.070	0.749 to 0.896	9/9
	FA1000	0.329	0.387	0.817	0.173 to 0.531	9/9	0.792	0.011	0.253	0.732 to 0.855	9/9
	FA2000	0.373	0.323	0.972	0.173 to 0.679	9/9	0.758	0.018	0.191	0.699 to 0.795	9/9
	MD1000	0.399	0.288	0.869	0.222 to 0.729	9/9	0.741	0.022	0.172	0.626 to 0.896	9/9
	MD2000	0.355	0.348	0.835	0.173 to 0.679	9/9	0.766	0.016	0.138	0.663 to 0.933	9/9
	RD1000	0.399	0.288	0.869	0.222 to 0.729	9/9	0.741	0.022	0.172	0.626 to 0.896	9/9
	RD2000	0.234	0.545	0.909	0.000 to 0.507	8/9	0.596	0.090	0.471	0.417 to 0.933	9/9
Precuneus	HFC	0.078	0.842	0.965	-0.222 to 0.371	7/9	-0.238	0.537	0.862	-0.614 to 0.049	8/9
	CSF	0.572	0.108	0.619	0.408 to 0.726	9/9	0.026	0.948	1.000	-0.405 to 0.160	6/9
	GM	0.139	0.722	0.965	-0.086 to 0.507	6/9	0.664	0.051	0.524	0.537 to 0.896	9/9
	WM	0.173	0.656	0.965	-0.074 to 0.395	7/9	0.094	0.811	0.965	-0.307 to 0.381	8/9
	AxD1000	0.381	0.311	0.828	0.222 to 0.815	9/9	0.077	0.845	0.948	-0.147 to 0.405	5/9
	AxD2000	0.321	0.400	0.915	0.148 to 0.741	9/9	-0.187	0.629	0.825	-0.405 to 0.061	8/9
	FA1000	0.321	0.400	0.817	0.099 to 0.556	9/9	-0.043	0.913	0.955	-0.442 to 0.209	4/9
	FA2000	-0.156	0.689	0.972	-0.531 to 0.000	7/9	-0.043	0.913	0.955	-0.442 to 0.209	4/9
	MD1000	0.312	0.414	0.869	0.124 to 0.717	9/9	-0.187	0.629	0.905	-0.405 to 0.061	8/9
	MD2000	0.251	0.514	0.835	0.049 to 0.642	9/9	-0.323	0.396	0.690	-0.488 to -0.110	9/9
	RD1000	0.251	0.514	0.869	0.049 to 0.642	9/9	-0.341	0.370	0.608	-0.512 to -0.110	9/9
	RD2000	0.182	0.639	0.909	-0.049 to 0.544	8/9	-0.341	0.370	0.608	-0.512 to -0.110	9/9
Lt Thalamus	HFC	-0.598	0.089	0.965	-0.776 to -0.457	9/9	0.281	0.464	0.862	0.110 to 0.577	9/9
	CSF	-0.191	0.623	0.982	-0.544 to 0.049	8/9	-0.051	0.896	1.000	-0.270 to 0.368	7/9
	GM	0.303	0.428	0.965	0.086 to 0.556	9/9	-0.221	0.567	0.870	-0.439 to -0.086	9/9
	WM	-0.043	0.912	0.965	-0.346 to 0.247	4/9	-0.026	0.948	0.965	-0.221 to 0.405	7/9
	AxD1000	-0.503	0.168	0.773	-0.630 to -0.346	9/9	0.434	0.243	0.559	0.184 to 0.872	9/9
	AxD2000	-0.503	0.168	0.915	-0.630 to -0.346	9/9	0.247	0.522	0.825	0.110 to 0.798	9/9
	FA1000	0.191	0.623	0.817	0.012 to 0.568	9/9	0.715	0.030	0.306	0.589 to 0.830	9/9
	FA2000	0.147	0.705	0.972	-0.099 to 0.296	7/9	0.732	0.025	0.191	0.663 to 0.807	9/9
	MD1000	-0.572	0.108	0.619	-0.717 to -0.445	9/9	0.264	0.493	0.809	0.135 to 0.822	9/9
	MD2000	-0.572	0.108	0.619	-0.729 to -0.445	9/9	0.077	0.845	0.883	-0.110 to 0.552	6/9
	RD1000	-0.633	0.068	0.518	-0.803 to -0.519	9/9	0.230	0.552	0.767	0.086 to 0.773	9/9
	RD2000	-0.572	0.108	0.819	-0.729 to -0.445	9/9	-0.085	0.828	0.906	-0.319 to 0.319	7/9
Rt Thalamus	HFC	0.078	0.842	0.965	-0.063 to 0.346	6/9	0.553	0.122	0.862	0.430 to 0.627	9/9
	CSF	-0.165	0.672	0.982	-0.494 to 0.099	7/9	0.043	0.913	1.000	-0.184 to 0.503	3/9
	GM	0.182	0.639	0.965	-0.086 to 0.408	7/9	0.281	0.464	0.870	0.147 to 0.439	9/9
	WM	0.113	0.773	0.965	-0.148 to 0.321	7/9	-0.247	0.522	0.965	-0.390 to 0.086	8/9

	AxD1000	-0.191	0.623	0.828	-0.358 to 0.049	8/9	0.570	0.109	0.417	0.381 to 0.933	9/9
	AxD2000	0.043	0.912	0.953	-0.185 to 0.272	5/9	0.306	0.423	0.825	0.196 to 0.884	9/9
	FA1000	-0.182	0.639	0.817	-0.469 to 0.086	7/9	0.170	0.661	0.955	-0.135 to 0.516	8/9
	FA2000	-0.156	0.689	0.972	-0.531 to 0.000	7/9	0.221	0.567	0.784	-0.061 to 0.589	8/9
	MD1000	-0.026	0.947	0.990	-0.272 to 0.272	5/9	0.477	0.194	0.447	0.246 to 0.933	9/9
	MD2000	0.113	0.773	0.982	-0.086 to 0.457	7/9	0.306	0.423	0.690	0.196 to 0.884	9/9
	RD1000	0.286	0.456	0.869	0.086 to 0.469	9/9	0.460	0.213	0.490	0.221 to 0.908	9/9
	RD2000	0.191	0.623	0.909	-0.049 to 0.420	8/9	0.179	0.645	0.781	0.012 to 0.700	9/9
MFG	HFC	-0.130	0.739	0.965	-0.457 to 0.136	6/9	0.119	0.760	0.862	0.000 to 0.491	7/9
	CSF	0.347	0.361	0.922	0.259 to 0.778	9/9	-0.298	0.436	1.000	-0.430 to 0.012	8/9
	GM	-0.269	0.485	0.965	-0.642 to -0.049	9/9	0.009	0.983	0.983	-0.356 to 0.282	3/9
	WM	0.035	0.929	0.965	-0.272 to 0.272	4/9	-0.315	0.409	0.965	-0.466 to -0.012	9/9
	AxD1000	0.121	0.756	0.828	-0.124 to 0.469	7/9	-0.034	0.931	0.948	-0.270 to 0.393	6/9
	AxD2000	0.121	0.756	0.915	-0.124 to 0.469	7/9	-0.034	0.931	0.931	-0.270 to 0.393	6/9
	FA1000	-0.477	0.195	0.817	-0.693 to -0.309	9/9	0.060	0.879	0.955	-0.147 to 0.405	5/9
	FA2000	-0.243	0.529	0.972	-0.630 to -0.124	9/9	0.392	0.297	0.570	0.233 to 0.700	9/9
	MD1000	0.121	0.756	0.869	-0.124 to 0.469	7/9	-0.017	0.965	0.983	-0.270 to 0.417	6/9
	MD2000	0.121	0.756	0.982	-0.124 to 0.469	7/9	-0.077	0.845	0.883	-0.331 to 0.331	7/9
	RD1000	0.121	0.756	0.869	-0.124 to 0.469	7/9	-0.017	0.965	0.965	-0.270 to 0.417	6/9
	RD2000	0.121	0.756	0.909	-0.124 to 0.469	7/9	-0.102	0.794	0.906	-0.393 to 0.295	8/9
IPL	HFC	0.156	0.689	0.965	-0.111 to 0.482	7/9	-0.094	0.811	0.862	-0.405 to 0.184	7/9
	CSF	0.459	0.214	0.702	0.272 to 0.642	9/9	-0.170	0.661	1.000	-0.331 to 0.074	8/9
	GM	0.026	0.947	0.965	-0.185 to 0.358	4/9	0.809	0.008	0.191	0.732 to 0.896	9/9
	WM	0.286	0.456	0.965	0.074 to 0.531	9/9	0.017	0.965	0.965	-0.160 to 0.466	2/9
	AxD1000	0.251	0.514	0.828	-0.012 to 0.531	8/9	-0.289	0.450	0.733	-0.491 to 0.000	7/9
	AxD2000	0.165	0.672	0.915	0.000 to 0.556	6/9	-0.519	0.152	0.533	-0.687 to -0.307	9/9
	FA1000	-0.182	0.639	0.817	-0.315 to 0.012	7/9	0.264	0.493	0.944	0.000 to 0.466	8/9
	FA2000	-0.035	0.929	0.972	-0.198 to 0.284	7/9	0.451	0.223	0.570	0.270 to 0.687	9/9
	MD1000	0.225	0.560	0.869	-0.049 to 0.494	8/9	-0.485	0.186	0.447	-0.638 to -0.258	9/9
	MD2000	0.234	0.545	0.835	-0.037 to 0.494	8/9	-0.460	0.213	0.545	-0.610 to -0.221	9/9
	RD1000	0.130	0.739	0.869	-0.173 to 0.358	7/9	-0.460	0.213	0.490	-0.610 to -0.221	9/9
	RD2000	0.165	0.672	0.909	-0.124 to 0.395	8/9	-0.383	0.309	0.592	-0.512 to -0.172	9/9
Lt Frontal Lobe	HFC	0.243	0.529	0.965	0.025 to 0.618	9/9	-0.434	0.243	0.862	-0.663 to -0.184	9/9
	CSF	0.208	0.591	0.982	-0.062 to 0.469	8/9	-0.230	0.552	1.000	-0.410 to -0.012	9/9
	GM	0.061	0.877	0.965	-0.247 to 0.259	7/9	0.255	0.507	0.870	0.000 to 0.503	8/9
	WM	0.061	0.877	0.965	-0.235 to 0.358	5/9	-0.315	0.409	0.965	-0.466 to -0.012	9/9
	AxD1000	0.130	0.739	0.828	-0.062 to 0.494	8/9	-0.111	0.777	0.948	-0.356 to 0.282	7/9
	AxD2000	0.130	0.739	0.915	-0.062 to 0.494	8/9	-0.153	0.694	0.840	-0.417 to 0.221	7/9
	FA1000	-0.182	0.639	0.817	-0.358 to 0.086	8/9	-0.085	0.828	0.955	-0.331 to 0.319	8/9
	FA2000	-0.139	0.722	0.972	-0.358 to 0.148	7/9	-0.085	0.828	0.906	-0.331 to 0.319	8/9
	MD1000	0.277	0.470	0.869	0.049 to 0.556	9/9	-0.111	0.777	0.983	-0.356 to 0.282	7/9
	MD2000	0.329	0.387	0.835	0.148 to 0.531	9/9	-0.238	0.537	0.747	-0.540 to 0.098	8/9
	RD1000	0.312	0.414	0.869	0.099 to 0.593	9/9	-0.153	0.694	0.887	-0.417 to 0.221	7/9

	RD2000	0.303	0.428	0.909	0.111 to 0.494	9/9	-0.281	0.464	0.705	-0.602 to 0.037	8/9
Rt Frontal Lobe	HFC	-0.130	0.739	0.965	-0.482 to 0.111	7/9	-0.247	0.522	0.862	-0.503 to 0.086	8/9
	CSF	-0.104	0.790	0.982	-0.457 to 0.037	6/9	-0.204	0.598	1.000	-0.366 to 0.025	8/9
	GM	-0.147	0.705	0.965	-0.469 to 0.124	7/9	0.043	0.913	0.983	-0.282 to 0.331	6/9
	WM	0.104	0.790	0.965	-0.185 to 0.358	7/9	-0.272	0.478	0.965	-0.466 to 0.049	7/9
	AxD1000	0.234	0.545	0.828	0.148 to 0.556	9/9	0.051	0.896	0.948	-0.147 to 0.516	4/9
	AxD2000	0.208	0.591	0.915	0.111 to 0.519	9/9	-0.077	0.845	0.931	-0.331 to 0.331	7/9
	FA1000	-0.243	0.529	0.817	-0.408 to 0.012	7/9	0.068	0.862	0.955	-0.122 to 0.540	6/9
	FA2000	-0.035	0.929	0.972	-0.272 to 0.284	6/9	0.085	0.828	0.906	-0.098 to 0.565	6/9
	MD1000	0.208	0.591	0.869	0.111 to 0.519	9/9	0.009	0.983	0.983	-0.209 to 0.454	3/9
	MD2000	0.243	0.529	0.835	0.111 to 0.556	9/9	-0.094	0.811	0.883	-0.356 to 0.307	8/9
	RD1000	0.243	0.529	0.869	0.111 to 0.556	9/9	-0.077	0.845	0.965	-0.331 to 0.331	7/9
	RD2000	0.243	0.529	0.909	0.062 to 0.420	9/9	-0.230	0.552	0.705	-0.528 to 0.110	8/9
Lt Limbic Lobe	HFC	-0.017	0.965	0.965	-0.136 to 0.296	7/9	-0.085	0.828	0.862	-0.246 to 0.319	7/9
	CSF	0.225	0.560	0.982	-0.012 to 0.457	7/9	-0.017	0.965	1.000	-0.196 to 0.295	5/9
	GM	-0.416	0.266	0.965	-0.605 to -0.210	9/9	0.238	0.537	0.870	-0.049 to 0.614	8/9
	WM	0.182	0.639	0.965	-0.062 to 0.395	7/9	-0.017	0.965	0.965	-0.220 to 0.258	6/9
	AxD1000	0.061	0.877	0.917	-0.185 to 0.408	6/9	0.579	0.102	0.417	0.393 to 0.708	9/9
	AxD2000	0.017	0.965	0.965	-0.247 to 0.346	5/9	0.409	0.275	0.702	0.147 to 0.530	9/9
	FA1000	-0.130	0.739	0.850	-0.315 to 0.074	8/9	-0.553	0.122	0.474	-0.724 to -0.356	9/9
	FA2000	0.156	0.689	0.972	0.012 to 0.544	9/9	-0.655	0.055	0.212	-0.781 to -0.503	9/9
	MD1000	0.165	0.672	0.869	-0.049 to 0.544	8/9	0.579	0.102	0.393	0.393 to 0.708	9/9
	MD2000	-0.009	0.982	0.982	-0.284 to 0.309	5/9	0.536	0.137	0.533	0.331 to 0.708	9/9
	RD1000	0.095	0.807	0.884	-0.148 to 0.445	6/9	0.579	0.102	0.471	0.393 to 0.708	9/9
	RD2000	-0.009	0.982	1.000	-0.284 to 0.309	5/9	0.536	0.137	0.484	0.331 to 0.708	9/9
Rt Limbic Lobe	HFC	0.234	0.545	0.965	0.012 to 0.605	9/9	0.162	0.678	0.862	0.048 to 0.552	9/9
	CSF	0.494	0.177	0.702	0.346 to 0.667	9/9	0.247	0.522	1.000	-0.012 to 0.479	8/9
	GM	-0.260	0.499	0.965	-0.544 to -0.012	9/9	0.085	0.828	0.983	-0.123 to 0.565	6/9
	WM	0.017	0.965	0.965	-0.284 to 0.259	4/9	-0.204	0.598	0.965	-0.325 to 0.147	7/9
	AxD1000	0.338	0.374	0.828	0.210 to 0.766	9/9	0.834	0.005	0.047	0.761 to 0.878	9/9
	AxD2000	0.321	0.400	0.915	0.198 to 0.754	9/9	0.766	0.016	0.092	0.663 to 0.855	9/9
	FA1000	-0.520	0.151	0.817	-0.756 to -0.371	9/9	-0.196	0.614	0.955	-0.491 to 0.160	8/9
	FA2000	0.130	0.739	0.972	-0.062 to 0.494	8/9	-0.306	0.423	0.748	-0.687 to 0.000	8/9
	MD1000	0.373	0.323	0.869	0.210 to 0.803	9/9	0.851	0.004	0.070	0.786 to 0.903	9/9
	MD2000	0.520	0.151	0.696	0.371 to 0.865	9/9	0.766	0.016	0.138	0.663 to 0.855	9/9
	RD1000	0.477	0.195	0.869	0.321 to 0.803	9/9	0.783	0.013	0.144	0.687 to 0.884	9/9
	RD2000	0.459	0.214	0.819	0.296 to 0.766	9/9	0.766	0.016	0.185	0.663 to 0.855	9/9
Lt Occipital Lobe	HFC	-0.026	0.947	0.965	-0.296 to 0.296	7/9	-0.315	0.409	0.862	-0.528 to -0.012	9/9
	CSF	0.468	0.204	0.702	0.284 to 0.630	9/9	-0.034	0.931	1.000	-0.282 to 0.086	5/9
	GM	0.260	0.499	0.965	0.025 to 0.519	9/9	0.545	0.129	0.524	0.393 to 0.724	9/9
	WM	0.364	0.336	0.965	0.161 to 0.655	9/9	0.136	0.727	0.965	-0.012 to 0.540	8/9
	AxD1000	0.425	0.255	0.828	0.235 to 0.576	9/9	-0.485	0.186	0.559	-0.638 to -0.258	9/9
	AxD2000	0.381	0.311	0.915	0.185 to 0.526	9/9	-0.502	0.168	0.533	-0.663 to -0.282	9/9

	FA1000	-0.329	0.387	0.817	-0.531 to -0.111	9/9	-0.638	0.064	0.369	-0.756 to -0.479	9/9
	FA2000	-0.199	0.607	0.972	-0.408 to 0.074	7/9	-0.664	0.051	0.212	-0.884 to -0.516	9/9
	MD1000	0.425	0.255	0.869	0.235 to 0.576	9/9	-0.485	0.186	0.447	-0.638 to -0.258	9/9
	MD2000	0.381	0.311	0.835	0.185 to 0.526	9/9	-0.502	0.168	0.533	-0.663 to -0.282	9/9
	RD1000	0.425	0.255	0.869	0.235 to 0.576	9/9	-0.460	0.213	0.490	-0.610 to -0.221	9/9
	RD2000	0.485	0.186	0.819	0.321 to 0.630	9/9	-0.502	0.168	0.484	-0.663 to -0.282	9/9
Rt Occipital Lobe	HFC	0.390	0.300	0.965	0.173 to 0.704	9/9	-0.255	0.507	0.862	-0.479 to 0.012	8/9
	CSF	0.598	0.089	0.619	0.457 to 0.701	9/9	0.068	0.862	1.000	-0.270 to 0.258	6/9
	GM	-0.156	0.689	0.965	-0.482 to 0.111	7/9	0.485	0.186	0.610	0.282 to 0.773	9/9
	WM	0.277	0.470	0.965	0.062 to 0.469	9/9	0.085	0.828	0.965	-0.147 to 0.491	7/9
	AxD1000	0.702	0.035	0.412	0.605 to 0.964	9/9	-0.196	0.614	0.882	-0.356 to 0.160	7/9
	AxD2000	0.676	0.046	0.525	0.568 to 0.927	9/9	-0.187	0.629	0.825	-0.516 to 0.172	7/9
	FA1000	-0.269	0.485	0.817	-0.457 to -0.037	9/9	-0.690	0.040	0.306	-0.830 to -0.552	9/9
	FA2000	-0.615	0.078	0.972	-0.819 to -0.469	9/9	-0.800	0.010	0.191	-0.892 to -0.712	9/9
	MD1000	0.676	0.046	0.525	0.568 to 0.927	9/9	-0.077	0.845	0.983	-0.356 to 0.331	7/9
	MD2000	0.676	0.046	0.525	0.568 to 0.927	9/9	-0.230	0.552	0.747	-0.577 to 0.110	8/9
	RD1000	0.650	0.058	0.518	0.531 to 0.889	9/9	-0.077	0.845	0.965	-0.356 to 0.331	7/9
	RD2000	0.650	0.058	0.669	0.531 to 0.889	9/9	-0.230	0.552	0.705	-0.577 to 0.110	8/9
Lt Parietal Lobe	HFC	0.130	0.739	0.965	-0.148 to 0.445	7/9	-0.392	0.297	0.862	-0.835 to -0.172	9/9
	CSF	0.260	0.499	0.982	0.000 to 0.395	7/9	-0.255	0.507	1.000	-0.390 to 0.000	8/9
	GM	-0.035	0.929	0.965	-0.235 to 0.284	6/9	0.621	0.074	0.524	0.479 to 0.835	9/9
	WM	0.191	0.623	0.965	-0.062 to 0.420	8/9	-0.145	0.710	0.965	-0.282 to 0.209	7/9
	AxD1000	0.269	0.485	0.828	0.000 to 0.556	8/9	0.289	0.450	0.733	0.147 to 0.737	9/9
	AxD2000	0.234	0.545	0.915	-0.037 to 0.494	8/9	0.247	0.522	0.825	0.086 to 0.675	9/9
	FA1000	0.026	0.947	0.965	-0.086 to 0.284	5/9	-0.009	0.983	0.983	-0.393 to 0.258	4/9
	FA2000	-0.199	0.607	0.972	-0.445 to 0.074	8/9	0.017	0.965	0.965	-0.184 to 0.319	4/9
	MD1000	0.191	0.623	0.869	-0.086 to 0.432	8/9	-0.128	0.743	0.983	-0.454 to 0.209	7/9
	MD2000	0.286	0.456	0.835	0.049 to 0.544	9/9	-0.289	0.450	0.690	-0.503 to -0.049	9/9
	RD1000	0.121	0.756	0.869	-0.173 to 0.334	7/9	-0.409	0.275	0.575	-0.537 to -0.196	9/9
	RD2000	0.321	0.400	0.909	0.099 to 0.544	9/9	-0.409	0.275	0.575	-0.537 to -0.196	9/9
Rt Parietal Lobe	HFC	0.182	0.639	0.965	-0.074 to 0.519	7/9	-0.306	0.423	0.862	-0.712 to -0.049	9/9
	CSF	0.425	0.255	0.732	0.222 to 0.741	9/9	0.060	0.879	1.000	-0.037 to 0.246	4/9
	GM	-0.052	0.894	0.965	-0.346 to 0.247	6/9	0.536	0.137	0.524	0.356 to 0.847	9/9
	WM	0.199	0.607	0.965	-0.062 to 0.482	7/9	-0.281	0.464	0.965	-0.479 to 0.012	7/9
	AxD1000	0.338	0.374	0.828	0.185 to 0.778	9/9	0.272	0.478	0.733	0.147 to 0.712	9/9
	AxD2000	0.338	0.374	0.915	0.185 to 0.778	9/9	0.187	0.629	0.825	0.025 to 0.589	9/9
	FA1000	-0.243	0.529	0.817	-0.445 to 0.012	8/9	0.392	0.297	0.684	0.184 to 0.602	9/9
	FA2000	-0.052	0.894	0.972	-0.259 to 0.272	7/9	0.417	0.264	0.570	0.221 to 0.602	9/9
	MD1000	0.338	0.374	0.869	0.185 to 0.778	9/9	-0.366	0.333	0.638	-0.537 to -0.135	9/9
	MD2000	0.321	0.400	0.835	0.148 to 0.741	9/9	-0.366	0.333	0.638	-0.537 to -0.135	9/9
	RD1000	0.286	0.456	0.869	0.111 to 0.704	9/9	-0.366	0.333	0.588	-0.537 to -0.135	9/9
	RD2000	0.286	0.456	0.909	0.049 to 0.544	9/9	-0.341	0.370	0.608	-0.512 to -0.135	9/9
	HFC	-0.078	0.842	0.965	-0.272 to 0.222	7/9	0.145	0.710	0.862	-0.135 to 0.430	8/9

Lt Temporal Lobe	CSF	0.052	0.894	0.982	-0.235 to 0.222	7/9	-0.094	0.811	1.000	-0.270 to 0.184	7/9
	GM	-0.191	0.623	0.965	-0.445 to 0.074	7/9	0.434	0.243	0.699	0.209 to 0.565	9/9
	WM	0.121	0.756	0.965	-0.148 to 0.321	7/9	-0.068	0.862	0.965	-0.195 to 0.295	7/9
	AxD1000	0.329	0.387	0.828	0.136 to 0.729	9/9	-0.153	0.694	0.939	-0.307 to 0.221	7/9
	AxD2000	0.095	0.807	0.928	-0.173 to 0.420	7/9	-0.179	0.645	0.825	-0.368 to 0.184	7/9
	FA1000	0.485	0.186	0.817	0.321 to 0.618	9/9	-0.358	0.345	0.721	-0.614 to -0.074	9/9
	FA2000	0.260	0.499	0.972	0.012 to 0.544	9/9	-0.400	0.286	0.570	-0.614 to -0.135	9/9
	MD1000	0.130	0.739	0.869	-0.136 to 0.457	7/9	-0.221	0.567	0.870	-0.405 to 0.123	8/9
	MD2000	-0.017	0.965	0.982	-0.334 to 0.259	5/9	-0.196	0.614	0.784	-0.368 to 0.160	7/9
	RD1000	0.130	0.739	0.869	-0.136 to 0.457	7/9	-0.221	0.567	0.767	-0.405 to 0.123	8/9
	RD2000	-0.121	0.756	0.909	-0.469 to 0.124	7/9	-0.264	0.493	0.705	-0.466 to 0.061	8/9
Rt Temporal Lobe	HFC	-0.130	0.739	0.965	-0.457 to 0.136	7/9	0.162	0.678	0.862	0.049 to 0.675	9/9
	CSF	-0.009	0.982	0.982	-0.321 to 0.148	2/9	0.000	1.000	1.000	-0.233 to 0.319	0/9
	GM	-0.260	0.499	0.965	-0.469 to -0.012	9/9	0.043	0.913	0.983	-0.331 to 0.331	7/9
	WM	0.087	0.825	0.965	-0.185 to 0.358	8/9	-0.119	0.760	0.965	-0.246 to 0.246	7/9
	AxD1000	0.225	0.560	0.828	0.025 to 0.618	9/9	-0.026	0.948	0.948	-0.147 to 0.405	7/9
	AxD2000	0.399	0.288	0.915	0.210 to 0.692	9/9	-0.043	0.913	0.931	-0.172 to 0.381	7/9
	FA1000	-0.017	0.965	0.965	-0.136 to 0.222	6/9	-0.162	0.678	0.955	-0.479 to 0.209	8/9
	FA2000	0.130	0.739	0.972	-0.136 to 0.371	8/9	-0.204	0.598	0.784	-0.540 to 0.147	8/9
	MD1000	0.225	0.560	0.869	0.025 to 0.618	9/9	-0.026	0.948	0.983	-0.147 to 0.405	7/9
	MD2000	0.295	0.442	0.835	0.099 to 0.692	9/9	-0.043	0.913	0.913	-0.172 to 0.381	7/9
	RD1000	0.295	0.442	0.869	0.099 to 0.692	9/9	-0.043	0.913	0.965	-0.172 to 0.381	7/9
	RD2000	0.295	0.442	0.909	0.099 to 0.692	9/9	-0.043	0.913	0.955	-0.172 to 0.381	7/9

Spearman rank correlation coefficients (rho, ϱ) are presented for the association between MMSE scores and MRI metrics (N=9). Adj.p-values were calculated using the Benjamini–Hochberg procedure within each MRI index separately for the before-treatment and after-treatment analyses. Leave-one-out (LOO) sensitivity analyses were performed by iteratively excluding one participant to evaluate the stability of the correlation. LOO sign consistency indicates the frequency (out of 9 iterations) with which the direction of the correlation remained unchanged. Abbreviation: anterior cingulate (AC), medial frontal gyrus (MFG), inferior parietal lobule (IPL), high-frequency conductivity (HFC); brain tissue volumes (BTV) of gray matter (GM), white matter (WM), and cerebrospinal fluid (CSF); diffusion indices of axial diffusivity (AxD), fractional anisotropy (FA), mean diffusivity (MD), and radial diffusivity (RD) with b=1000 and 2000.

3. Results of Voxel-based Analyses

Voxel-Based Analysis

The voxel-based paired T-test was performed to evaluate the difference in each map between before and after treatment in each group. Second, the voxel-based multiple regression analyses were performed to examine the associations between each map and MMSE scores. A significance level of $\alpha = 0.002$ was applied without correction for multiple comparisons and clusters with at least 100 contiguous voxels because the number of participants in each group was limited. We did not use p=0.001 because the number of participants in each group was 3 and the statistical power was very limited.

Results of the paired T-test of MRI maps between the after and before treatment

Supplementary Figure S2 shows the results of the voxel-based paired T-test of HFC and brain tissue volume maps between the after and before treatment. HFC in the 50cGy LDRT group was significantly higher after than before LDRT (Fig.S2A). HFC in the 4cGy LDRT group was significantly higher after than before LDRT (Fig.S2C). In voxel-based

analyses, HFC increased post-LDRT in multiple cortical and subcortical regions, including the right fusiform gyrus and right medial frontal gyrus, especially in the 50 cGy group. CSFV in the 50cGy LDRT group was significantly higher after than before LDRT (Fig.S2B). All other maps were not significantly different between the after and before LDRT. The detailed locations of the significant difference for the voxel-based paired T-test are listed in Supplementary Table S3.

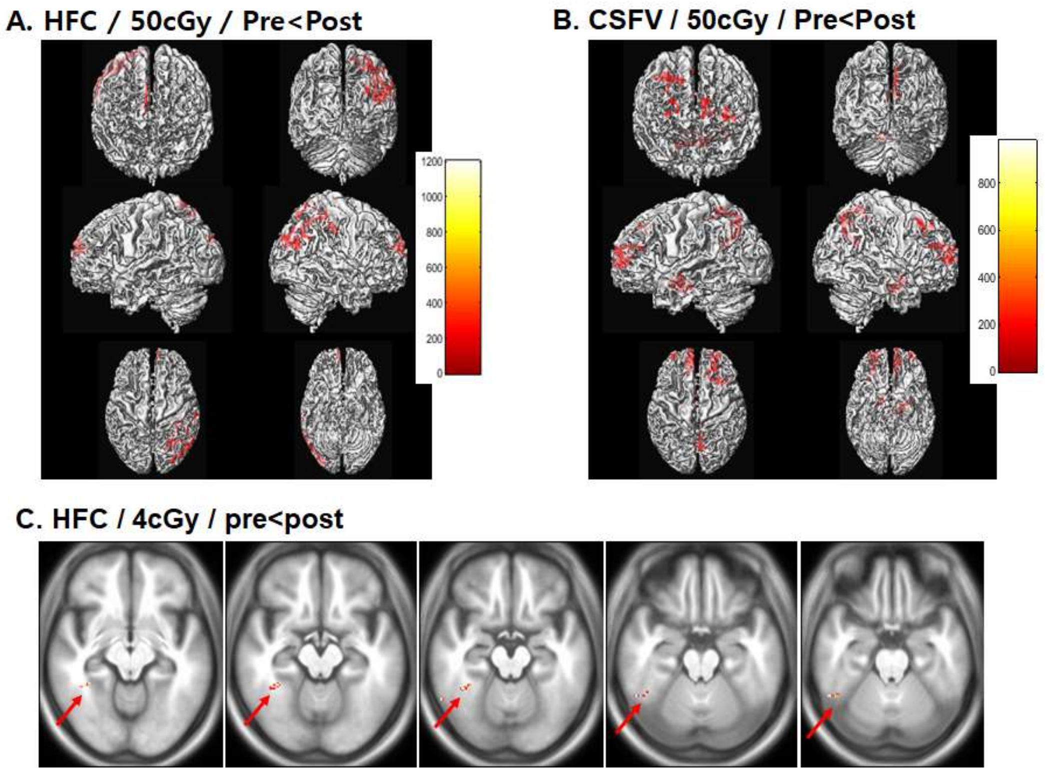

Supplementary Figure S2. Voxel-based paired t-test results comparing pre- and post-treatment maps for high-frequency conductivity (HFC) and cerebrospinal fluid (CSF) volume (CSFV). (A, C) Significant HFC increases were observed in the 50 cGy and 4 cGy groups, respectively. (B) Significant CSFV increases in the 50 cGy group. Results are shown at an uncorrected threshold of α=0.002 due to the small sample size. The detailed locations of the significant association areas are listed in Supplementary Table S3.

Supplementary Table S3. Detailed locations of the significant difference for the voxel-based paired T-test between the after and before treatment of high-frequency conductivity (HFC) and cerebrospinal fluid (CSF) tissue volume map

Group analysis	Cluster size	Cluster location	BA	Talairach coordinates			Z score	ROI
HFC with 4cGy: pre<post (Fig.S2A)								
	113	Rt Temporal Fusiform Gyrus	37	39.33	-46.52	-15.68	4.04	ROI
		Rt Posterior Declive		50.49	-50.25	-21.25	3.97	
		Rt Temporal Fusiform Gyrus	37	30.98	-41.15	-12.61	3.73	ROI
HFC with 50cGy: pre<post (Fig.S2C)								
	183	Rt Medial Frontal Gyrus	10	5.91	55.71	21.82	4.73	ROI
	1199	Rt Superior Parietal Lobule	7	20.24	-60.99	61	4.56	
		Rt Middle Temporal Gyrus	39	54.01	-64.63	24.74	4.54	
	345	Rt Inferior Parietal Lobule	40	53.83	-53.62	42	4.24	ROI
		Rt Parietal Sub-Gyral		32.95	-37.52	51.28	4.16	
		Rt Inferior Parietal Lobule	40	56.69	-33.81	41.22	3.99	ROI
CSF with 50cGy: pre<post (Fig.S2B)								
	317	Rt Parietal Precuneus		2.36	-69.24	45.05	4.88	ROI
		Rt Occipital Cuneus	7	3.93	-66.55	31.82	4.15	ROI
		Rt Limbic Posterior Cingulate	31	5.49	-52.93	22.33	4.07	
	281	Lt Limbic Anterior Cingulate		-5.22	39.13	18.71	4.57	ROI
		Lt Medial Frontal Gyrus		-10.89	39.64	28.12	4.32	ROI
	247	Lt Sub-lobar Lentiform Nucleus		-23.16	-6.59	-3.5	4.54	

		Lt Sub-lobar Extra-Nuclear		-30.02	-4.63	-8.83	4.11	
	152	Rt Superior Frontal Gyrus		21.07	41.14	26.1	4.36	
		Rt Frontal Sub-Gyral		23.82	29.94	25.08	3.93	
	176	Rt Medial Frontal Gyrus		13.84	21.18	43	4.34	ROI
		Rt Middle Frontal Gyrus		38.75	13.67	46.77	4.23	
		Rt Superior Frontal Gyrus		15.21	29.29	46.5	3.97	
	114	Lt Middle Frontal Gyrus		-28.7	51.09	12.69	4.33	
		Lt Superior Frontal Gyrus	10	-23.13	59.44	13.57	3.59	

The results of the voxel-based paired T-test between the after and before treatment of high-frequency conductivity (HFC) and cerebrospinal fluid (CSF) tissue volume maps are shown in Supplementary Figure S2.

Results of the multiple regression test of MRI maps with MMSE scores

The results of the voxel-based multiple regression test of HFC and FA maps with MMSE scores are shown in Supplementary Figure S3. The detailed locations of the significantly associated areas are listed in Supplementary Table S4. HFC after RT was negatively associated with MMSE scores. FA with b=1000 and 2000 before RT was negatively associated with MMSE scores.

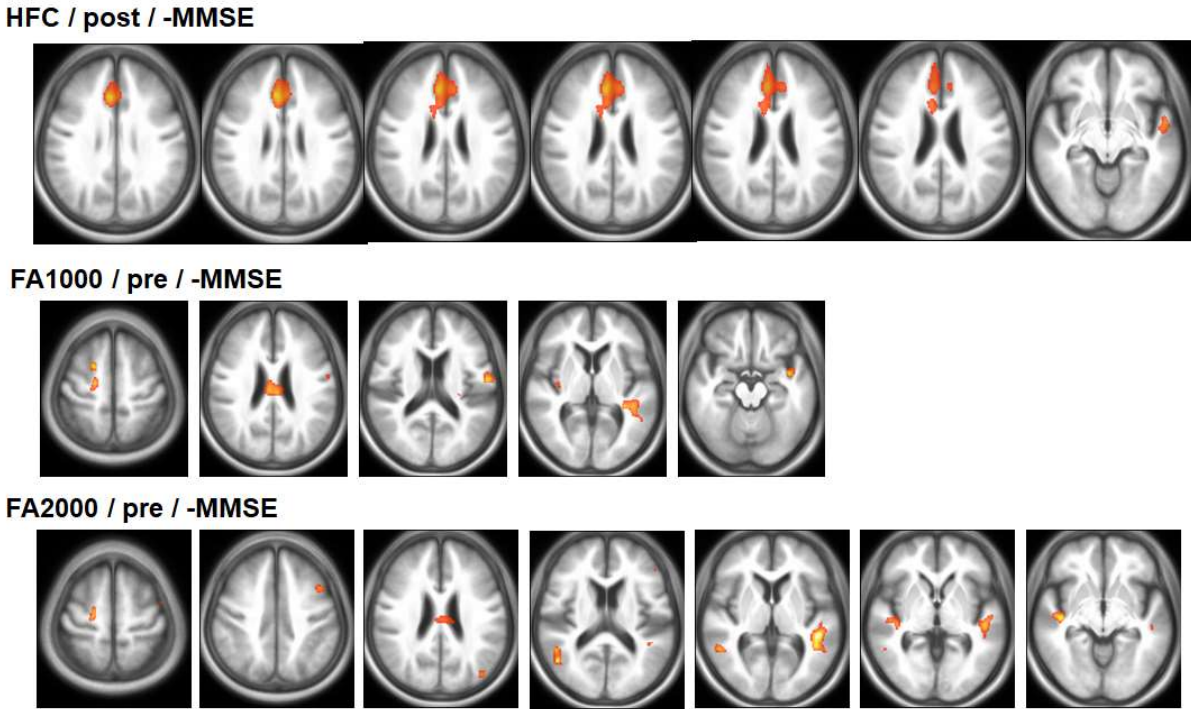

Supplementary Figure S3. Exploratory voxel-based multiple regression analysis between Mini-Mental State Examination (MMSE) scores and MRI metrics (high-frequency conductivity (HFC) and fractional anisotropy (FA). Post-treatment HFC showed a negative association with MMSE scores, while pre-treatment FA (at b=1000 and 2000 s/mm^2) was negatively associated with MMSE scores. The detailed locations of the significant association areas are listed in Supplementary Table S4.

Supplementary Table S4. Detailed locations of significant association areas from the voxel-based multiple regression between MRI images and Mini-Mental State Examination (MMSE) scores in all participants (untreated and treated) before and after treatment, separately

Group analysis	Cluster size	Cluster location	BA	Talairach coordinates			Z score	ROI
**HFC after RT: (-)MMSE**								
	276	Rt Medial Frontal Gyrus		3.75	-14.4	61.08	3.72	ROI
		Rt Frontal Precentral Gyrus		10.61	-19.15	66.15	3.51	
	1432	Rt Limbic Cingulate Gyrus		5.65	20.87	32.02	3.70	

	Lt Limbic Cingulate Gyrus		-6.8	25.39	29.54	3.20	
	Rt Limbic Anterior Cingulate		7.08	10.2	25.63	3.19	ROI
234	Lt Superior Temporal Gyrus	38	-50.85	-1.86	-7.57	3.37	
**FA1000 before RT: (-)MMSE**							
372	Lt Parietal Postcentral Gyrus		-55.37 -11.31 17.13			3.90	
229	Lt Temporal Sub-Gyral		-38.31 -2.93 -11.51			3.86	
157	Rt Frontal Sub-Gyral		17.79 -2.38 53			3.74	
611	Rt Limbic Cingulate Gyrus	23	5.58 -13.94 27.37			3.70	
674	Lt Temporal Sub-Gyral		-41.43	-41.08	3.74	3.67	
	Lt Superior Temporal Gyrus		-45.5	-26.69	0.98	3.59	
128	Lt Superior Frontal Gyrus		-22.13	46.91	40.78	3.63	
127	Rt Medial Frontal Gyrus		16.36	-17.74	51.52	3.56	ROI
174	Rt Sub-lobar Insula		40.69	-16.57	-4.71	3.53	ROI
	Rt Sub-lobar Claustrum		32.18	-5.52	12.41	3.15	
	Rt Sub-lobar Extra-Nuclear		33.61	-14.78	6.15	3.07	
**FA2000 before RT: (-)MMSE**							
1115	Lt Temporal Sub-Gyral		-44.19	-36.87	4.09	4.34	
	Lt Superior Temporal Gyrus		-45.49	-26.56	-0.36	3.65	
409	Rt Middle Temporal Gyrus		44.5	-60.34	10.12	4.07	
379	Rt Sub-lobar Insula		39.3	-17.96	-4.87	3.90	ROI
	Rt Sub-lobar Extra-Nuclear		36.41	-25.59	1.12	3.34	
135	Lt Temporal Sub-Gyral		-34.11	-1.29	-13.99	3.84	
105	Lt Inferior Frontal Gyrus	46	-48.24	28.44	14.26	3.66	
158	Lt Middle Temporal Gyrus		-41.68	-71.73	14.34	3.62	
127	Rt Frontal Sub-Gyral		17.7	-20.81	53.95	3.54	
154	Lt Inferior Parietal Lobule		-30.9	-48.11	46.49	3.40	ROI
123	Lt Middle Frontal Gyrus		-41.72	7.49	40.77	3.36	
244	Lt Sub-lobar Extra-Nuclear		-8.31	-23.52	24.88	3.18	
	Rt Limbic Cingulate Gyrus	23	5.56	-14.07	28.71	2.97	
116	Lt Frontal Precentral Gyrus		-41.89	-10.19	48.55	3.17	

Abbreviations: Rt, right; Lt, left; BA, Brodmann area; high-frequency conductivity (HFC), fractional anisotropy (FA) with b-value=1000 or 2000, Mini-Mental State Examination (MMSE), radiation treatment (RT)

Reference

Kim, D.-Y., Kim, J.S., Seo, Y.-S., Park, W.-Y., Kim, B.H., Hong, E.-H., Kim, J.Y., Cho, S.-J., Rhee, H.Y., Kim, A., Kim, K.Y., Oh, D.J., Chung, W.K., 2023. Evaluation of Efficacy and Safety Using Low Dose Radiation Therapy with Alzheimer's Disease: A Protocol for Multicenter Phase II Clinical Trial. Journal of Alzheimer's Disease 95, 1263-1272.
